# Solar cyclic variability can modulate winter Arctic climate

**DOI:** 10.1038/s41598-018-22854-0

**Published:** 2018-03-20

**Authors:** Indrani Roy

**Affiliations:** University of Exeter, Exeter, EX4 4QE UK

## Abstract

This study investigates the role of the eleven-year solar cycle on the Arctic climate during 1979–2016. It reveals that during those years, when the winter solar sunspot number (SSN) falls below 1.35 standard deviations (or mean value), the Arctic warming extends from the lower troposphere to high up in the upper stratosphere and vice versa when SSN is above. The warming in the atmospheric column reflects an easterly zonal wind anomaly consistent with warm air and positive geopotential height anomalies for years with minimum SSN and vice versa for the maximum. Despite the inherent limitations of statistical techniques, three different methods – Compositing, Multiple Linear Regression and Correlation – all point to a similar modulating influence of the sun on winter Arctic climate via the pathway of Arctic Oscillation. Presenting schematics, it discusses the mechanisms of how solar cycle variability influences the Arctic climate involving the stratospheric route. Compositing also detects an opposite solar signature on Eurasian snow-cover, which is a cooling during Minimum years, while warming in maximum. It is hypothesized that the reduction of ice in the Arctic and a growth in Eurasia, in recent winters, may in part, be a result of the current weaker solar cycle.

## Introduction

The last few decades have witnessed profound changes in the Arctic^1^, with the decline of perennial sea ice among the most striking. Arctic sea ice has declined by more than 40% since the late 1970s, and the 10 lowest September extents have all occurred within the last 10 years^2^. Studies suggest that 50–60% of that ice loss is likely caused by externally forced anthropogenic emissions^3–5^ with the rest caused by natural climate variability^6^. The pathways for natural variability are via the interplay among polar vortex, mid-latitude jet and planetary waves^7,8^. This study focuses on an advanced understanding of solar cyclic variability on Arctic climate via stratospheric pathways.

The sun is the primary source of energy in the earth/atmosphere system, but the actual role of the sun and related mechanisms to support varied regional climate responses and its seasonality around the world, are still poorly understood^1^. Solar energy output varies in cycles, of which the 11-year cyclic variability is one of the most crucial ones. It causes differences in the amount of solar energy absorbed in the UV part of the spectrum within the upper stratosphere, varying from 6 to 8%^9^. Such variation is believed to be one of the most important solar energy outputs to influence the climate of the earth and that knowledge of cyclic behaviour can also be used for future prediction purposes. Apart from solar UV related effects on earth’s climate^9,10^, studies also identified effects related to solar particle precipitation^11,12^.

During normal boreal winter (December, January and February (DJF)), there is a general warming in the upper stratosphere of Southern Hemisphere (SH) (being summer season) compared to Northern Hemisphere (NH) (being winter season). The temperature difference between the northern high and mid-latitudes generates upper stratospheric polar vortex features and associated jet in the NH (DJF), following well-known thermal wind balance relationship. The polar vortex in hemispheric winter season is generated by a band of upper-level winds (in the form of a jet) that circulate the pole in both hemispheres around the upper stratosphere and plays a major role in regulating the temperature of polar surfaces^13^. During active solar years of DJF, there is an enhanced warming in the upper stratosphere of SH, and subsequently stronger temperature contrast, which causes stronger upper stratospheric polar vortex features (NH) and stronger jet. Planetary waves originating from the troposphere can propagate through NH westerlies, and longer waves (wave number ~1–3) can reach up to upper stratosphere. During an inactive phase of solar years, they can easily break weaker jet and warm upper polar stratosphere. Jet being stronger in active solar years^14^, cannot be disrupted by planetary waves, following Charney-Drazin criteria. Thus air within the vortex is constricted inside to cause a cold polar vortex. A dynamical mechanism was also proposed to support a solar influence around tropical lower stratosphere^14^, involving upper stratospheric jet and upwardly propagating planetary waves. During active years, planetary waves being unable to pass through stronger jet deposit their momentum on the poleward side of the jet causing reduction in strength of Brewer-Dobson circulation, which subsequently warm tropical lower stratosphere^14^. Warming of the lower tropical stratosphere in active solar years could further lead to a response towards mid-latitude of the troposphere, modulating Ferrel cell^15^, by weakening it alongside shifting it pole-wards.

Disturbances in the hemispheric winter polar vortex are shown to be strongly linked with polar annular modes^13^, (such as the Northern Annular Mode (NAM) in NH, and Southern Annular Mode (SAM) in Southern Hemisphere (SH)). Those represent a barometric see-saw pattern between the polar region and mid-latitudes in both hemispheres, extending from the upper stratosphere down to the troposphere and surface. The surface signature of NAM is commonly known as the Arctic Oscillation (AO). Anomalies in geopotential heights around the upper stratospheric polar vortex have been shown to extend towards the surface on a seasonal scale^13^.

Though the sun could be a potential driving factor to modulate Polar vortex features (e.g. strength of the jet, temperature)^10,14^ in the upper stratosphere, the phase of the El Niño Southern Oscillation (ENSO) and the Quasi-Biennial Oscillation (QBO) also appears to be important contributors. Links between ENSO and the upper polar vortex temperature have been documented in several studies^16,17^; during winter, warm-ENSO years result in a significantly warmer upper stratosphere in the NH polar and mid-latitudes^16^. The QBO and sun relationship appears more complex^18,19^. Warm polar upper stratospheric temperatures tend to occur during the west phase of QBO at solar maximum and also during the easterly phase at solar minimum, whereas, the upper stratosphere is colder during westerly QBO at solar minimum and during easterly QBO at solar maximum^18^. However, for QBO Ely in the solar Max phase, studies indicated a controversy and showed it is also warm^20^. Later it was reconciled^21^ that suggested it is the QBO height which caused the difference. Using QBO height 30 hPa indeed shows warm polar upper stratosphere for both the solar phases, Max as well as Min^20,21^. Various studies have also detected an influence of the El Nino Southern Oscillation (ENSO)^22^ and the Pacific Decadal Oscillation (PDO)^23^ on Arctic sea ice. An association between the sun and ENSO are discussed in various research^24–26^. Because of related complexities along with various linear and nonlinear couplings among major modes of variability, the role of the sun on Arctic air temperatures and sea ice extent and related mechanisms remains poorly understood/explored.

Recent Arctic Sea ice extent is declining at a faster pace than climate models projected^27^. The trends in surface and lower tropospheric air temperatures are also shown closely linked with sea ice decline^28^. A study that focused on 2007–2012 even indicated largest dynamic forcing on sea ice loss compared to any previous periods^29^. Presently, the Arctic is witnessing rapid sea ice loss, with the lowest maximum winter extents recorded in 2015 and 2016. Sea ice extent thus far in 2017 is also tracked at record low levels. While many studies point to anthropogenic influences on the long-term sea ice decline, this study is motivated by the potential links between the sun and the surface climate through stratospheric processes. Alongside warming in the Arctic, a cooling is noticed around Eurasian sector despite continuing rise of greenhouse gas concentrations^30^. Various modelling groups, however, made unsuccessful efforts to detect an association between Eurasian cooling and Arctic sea-ice decline^30^. In this work, we evaluate the impact of the solar 11-year cycle, measured in terms of solar sunspot number (SSN), as a driving factor to modulate Arctic and surrounding climate. The influences of SSN on various surface parameters, such as Sea Level Pressure (SLP)^25,31,32^, Sea Surface Temperature (SST)^25,26,32^, and the polar stratosphere are well recognised^18,21^. If there is indeed a link between the solar cycle and Arctic climate, it is possible that the 11-year solar cycle can be used to improve seasonal and decadal predictions of sea ice.

In the present study, we use a combination of observational and reanalysis datasets to uncover relationships between the sun’s variability and Arctic surface climate, via the modulation of NAM and downward propagation of anomaly from upper stratospheric winter polar vortex.

## Results

### Winter Arctic sea-ice extent during Solar Max and Min years and air temperature trend

**Figure 1:**
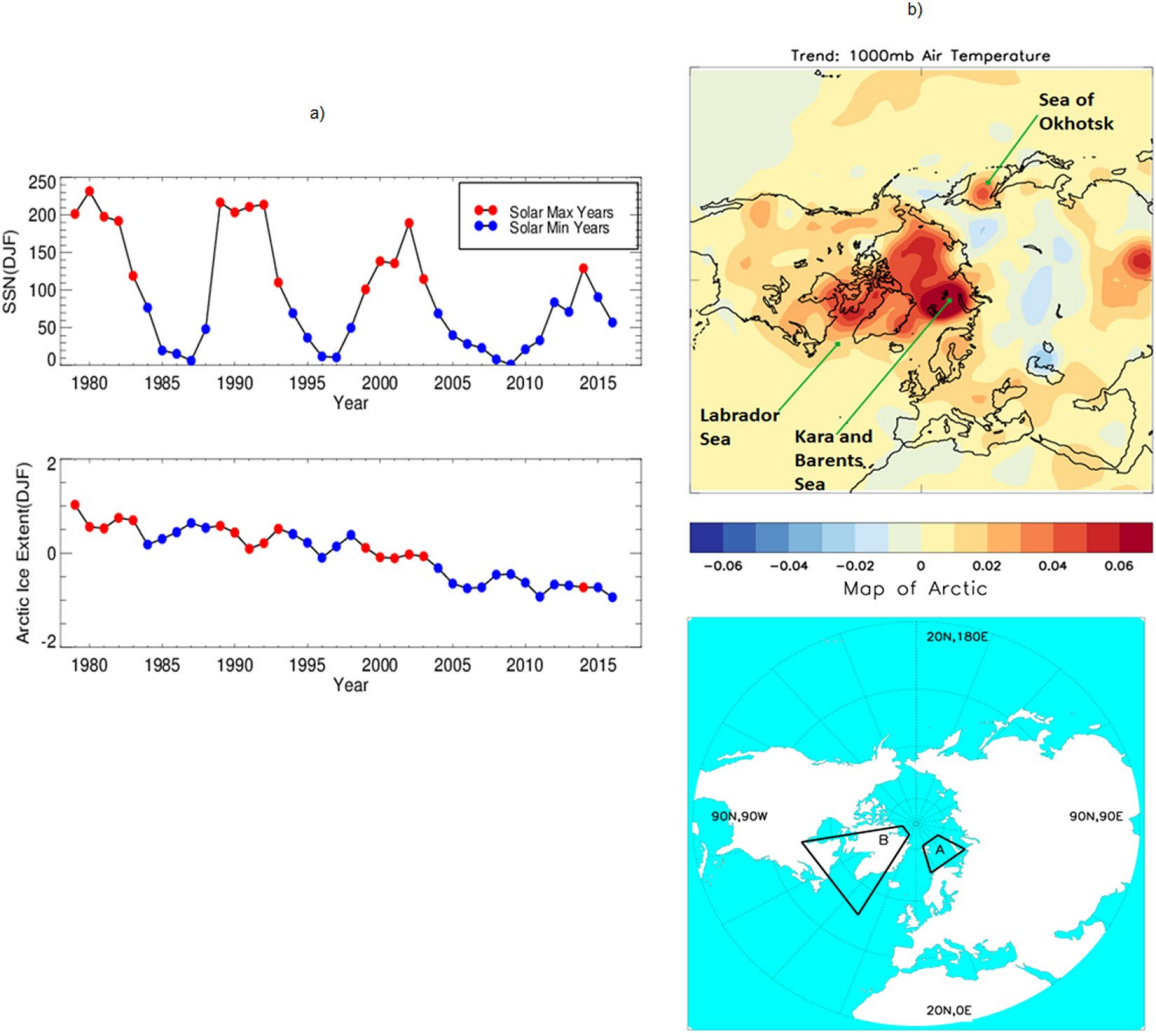
Time series plot (**a**) of SSN (DJF), [**a**, top panel] and Arctic sea-ice extent (DJF) anomaly (million sq-km) with respect to mean [**a**, bottom panel] during 1979–2016. The trend of winter air temperature (1000 mb) is also shown (**b**, top panel). In a map of Arctic (**b**, bottom panel) a region around Barents sea is marked by ‘A’ and a region around Labrador sea is marked by ‘B’. Plots are prepared using IDL version 8 (for A) and Met Office IDL (MIDL) (for B) software.

The extent of Arctic sea ice during winter is currently declining at a rate of about 3% per decade. Superimposed on that long-term trend are periods of higher and lower sea ice extent. Alongside a decline in the winter sea ice extent (Fig. 1a), there is also a long-term declining trend of the solar eleven-year cycle. Table 1 shows years of solar Max and Min with solar cycle numbers and those years are marked in Fig. 1a. From 1979 to 2003, there have been five SSN Max (red) and five Min years (blue) each. After 2003, we observe a total of 12 Min SSN years, and only one Max year of 2014 (Fig. 1a, top panel). Note, however, that while total sea ice extent has been declining, the locations of ice losses are mostly within the Kara and Barents seas, and within the Sea of Okhotsk, and to a lesser extent within the Labrador Sea. Those features are also detected in winter Arctic air temperature (1000 mb) trends in Fig. 1b (top). The trends in surface air temperatures are known to be closely linked with sea ice decline^28^. Studies suggest^33,34^ the overall winter sea ice variation in the Arctic results largely from the retreat of ice cover in the Barents Sea. The winter Barents Sea ice extent decline in recent decades has primarily been driven by heat transport variations in the ocean^33–35^. To put additional focus on that region we marked a region (70°N–81°N, 15°E–60°E) around Barents Sea by ‘A’ in Fig. 1b (bottom), as this location also received attention in previous works^35,36^. To analyse further on winter sea ice, we also chose an arbitrary area nearby (50°N–85°N, 80°W–30°W) covering a location of Labrador Sea and Greenland and marked it by ‘B’. We additionally examined another region (70°N–81°N, 80°W–60°E) which covered the longitude band of the whole of A and B, but only considered the overlapping latitude band of those two regions and termed as ‘A + B’.

**Table 1:** Years used for Solar Max and Min compositing. Few years from the beginning of the cycle 21 are excluded as period considered is 1979–2016.

**Solar Cycle Number**	**Cycle Year**	**Solar Minimum Years (<SSN 100)**	**Solar Maximum Years(>SSN 100)**
21	1976–1986	1984, 1985, 1986	1979, 1980, 1981, 1982, 1983
22	1986–1996	1987, 1988, 1994, 1995, 1996	1989, 1990, 1991, 1992, 1993
23	1996–2007	1997, 1998, 2004, 2005, 2006, 2007	1999, 2000, 2001, 2002, 2003
24	2008–2016	2008, 2009, 2010, 2011, 2012, 2013, 2015, 2016	2014
Total year (1979–2016)	38	22	16

### Solar signals in Arctic surface climate and vertical structure around the Troposphere

**Figure 2:**
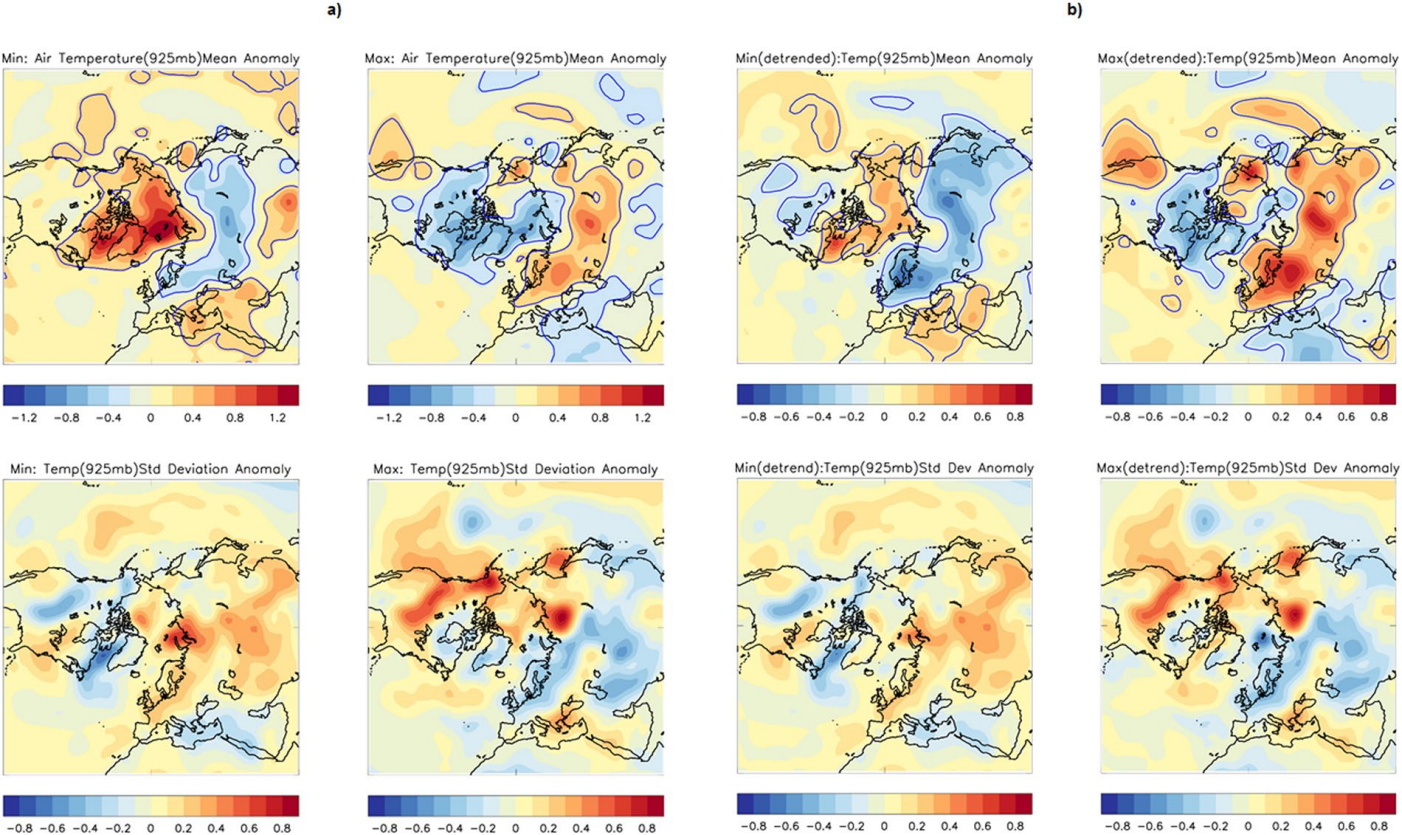
The Composite anomaly of winter Arctic air temperature (°C) at 925 mb, Mean and Standard Deviation for Solar Min (left) and Max (right) years. Results without detrending (**a**) and with detrending data (**b**) are presented. The base period is 1981–2010, and blue contour (top) marks significant regions up to 95% levels of mean differences. Plots are prepared using Met Office IDL (MIDL) software and top plots are also verified with the plots generated by the NOAA/OAR/ESRL PSD, Boulder, Colorado, USA, from their website at (http://www.esrl.noaa.gov/psd/).

Our initial work focused on composite technique. During winters with solar minima, DJF air temperatures at the 925 mb level are warmer than average over most of the Arctic Ocean, with the largest positive anomalies occurring over the Kara and the Barents Seas (Fig. 2, top left), this is consistent with declines in the winter sea ice cover within this region (also in agreement with Fig. 1b(top)). Warmer than average air temperatures are also observed in the Sea of Okhotsk and the Labrador Sea, again consistent with reduced sea ice cover. Cooling, on the other hand, is dominant over Siberia and Scandinavia (also seen in Fig. 1b(top)).

This warming/cooling pattern is reversed during solar Max years (Fig. 2, top right). The opposite cooling and warming signature around the Eurasian sector during solar Min and Max years, respectively, are also noted, which corresponds with warm Arctic/cold continent phenomena^30^. Contrasting the previous study that suggests such an association could be unrelated^30^, the current work points both the features could have a decadal connection. As a different signed signature in the last month/season could have a nullifying effect on winter Arctic sea ice extent, we checked whether it is also of a similar signed during previous other seasons. A significant warming pattern of the Arctic during Min (Fig. S1(a)) and cooling during Max (Fig. S1(b)) years is clearly evident in all those plots. The results from November, which is the previous month of DJF, also suggest similarly. The signature is comparatively weak during JJA.

While a general warming pattern over the Arctic Ocean emerges during solar Min years, the composite hides variability in the spatial pattern of the warming. The standard deviation shows less variability around places covering Greenland and the Labrador Sea (region marked by ‘B’) in both solar phases (Fig. 2a, bottom panel). The significant region for mean deviation of air temperature is outlined by blue (Fig. 2a, top panel) that takes into account the standard deviation of both the composite and climatology years. Local influences on Arctic air temperatures, as well as other modes of atmospheric variabilities – such as ENSO, the PDO, and the QBO – can modulate the temperature response on a year-to-year basis. A composite analysis can still detect an influence of the overall period of analysis that is due to the sun, in spite of the presence of other influences.

In case, trend influences the major results of compositing, we repeated the same analyses, de-trending the data beforehand. The purpose is to strengthen the robustness of any detected signal. After removing the trend, the signal around the Arctic region is seen to be still present for air temperature (925 mb) (Fig. 2b). With and without the trend, the standard deviation for both Max and Min indicates great similarity. It suggests the least around the region ‘B’ (region marked in Fig. 1b, bottom). The mean temperature of Eurasian snow cover practically suggests the same after detrending. Interestingly, the solar signal reduced substantially for region ‘A’, after detrending the data. The variation in region A is more for solar max years. It is consistent with previous works, which identified a stronger influence of the trend in the locations of Barents sea^33–36^.

The signal in the region ‘B’ after detrending reduced nearly half the magnitude of the actual signal (in places ~0.6 °C after detrending, which was 1.2 °C before detrending) but still significant (note a reduced colour scale). Since a relatively large signal is noticed compared to other locations even after detrending, it could be an explanation why we chose that location of region B. Whether oceanic longer-term influence could be another dominating factor to mask solar related signal on air temperature, when detrending the data, needs to be explored further. One interesting finding here relates to a robust solar influence on Eurasian snow cover. It is entirely unaffected by any trend related issues, which is probably due to the absence of any ocean-related variability in that location.

**Figure 3:**
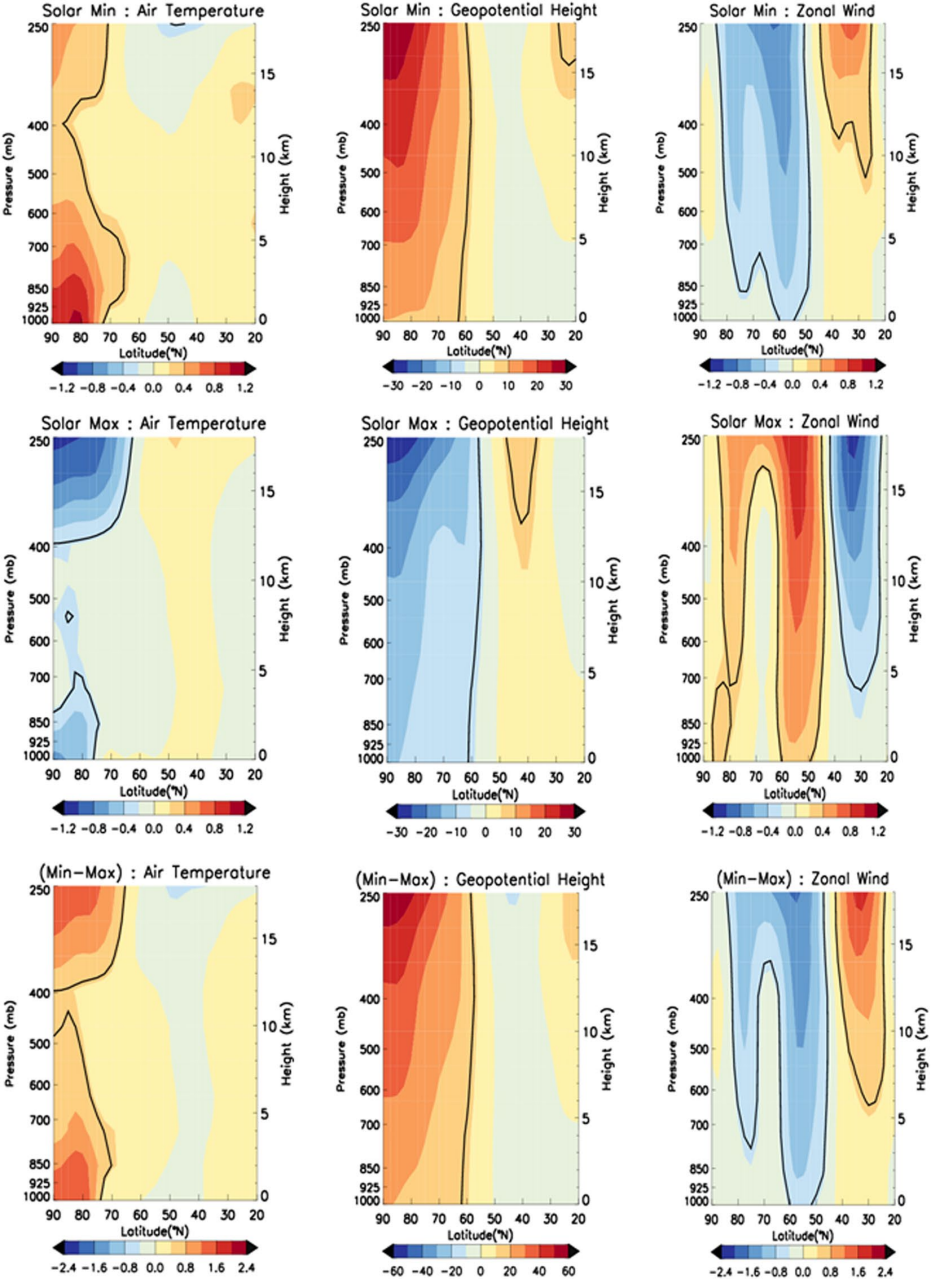
Latitude-Height (1000 mb up to 250 mb) plot for composites of air temperature (left), geopotential heights (middle) and zonal wind (right) during solar Min (top), solar Max (middle) and solar Min minus solar Max years (bottom). Levels usually significant up to 95% level are overlaid by the black coloured contour. Plots are generated using IDL software version 8 with the data from NOAA/OAR/ESRL PSD, Boulder, Colorado, USA, from their website at (http://www.esrl.noaa.gov/psd/).

While the warm Arctic/cold continent pattern is evident during solar Min years at the surface, warming also extends from the lower atmosphere towards the tropopause (Fig. 3). The vertical profile of positive temperature anomalies between 65°N and 90°N is clearly distinguished, with the strongest signature poleward of 75°N. Note also the largest warming occurs at the surface, as a result of enhanced exchanges of sensible and latent heat flux from the open ocean to the atmosphere during low sea ice years^28,37^. Consistent with positive air temperature anomalies, the vertical profile of geopotential height and zonal wind indicates positive and negative anomalies, respectively, from mid-latitudes towards the pole. Solar Max years (Fig. 3, middle row) show an opposite signature, with cooling throughout most of the troposphere poleward of 75°N, negative geopotential height anomalies and positive zonal wind anomalies. The signature of geopotential height and the zonal wind is strongest in the tropopause and decreases as we go down up to the surface. These differences between solar Min and Max years are further highlighted by the difference plots (bottom row, Fig. 3). The signature is similar with or without de-trending the data (Fig. 3 shows without de-trending).

### Solar signature captured in the polar vortex ‘Annular Features’ from the upper stratosphere down to the lower troposphere

**Figure 4:**
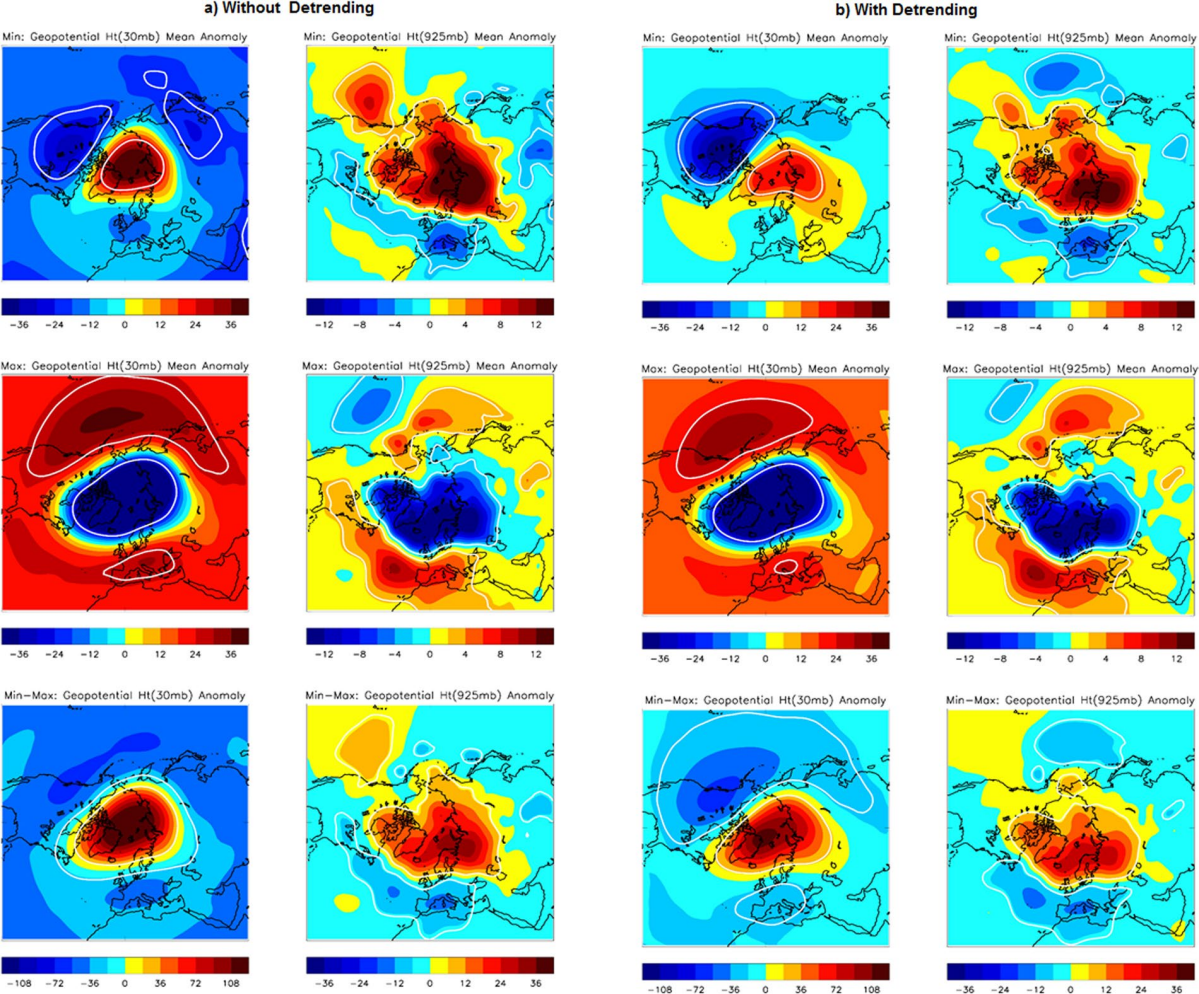
Geopotential height composites (**a**) without detrending and (**b**) with detrending data suggests a similar signature around the north pole in the upper stratosphere (30 mb, left) and at the surface (925 mb, right) for solar Min (top row) and solar Max years (middle row) respectively. The third row shows respective differences between Min and Max years. Significant regions up to 95% level are marked by the white contour. Plots are prepared using Met Office IDL (MIDL) software and also verified with the plots generated by the NOAA/OAR/ESRL PSD, Boulder, Colorado, USA, from their website at (http://www.esrl.noaa.gov/psd/).

Comparing the composite anomalies of geopotential heights for solar Min (top) and Max (middle) years in Fig. 4(a,b), it is clear that annular mode features are similar in the upper stratosphere (left) as well as on the surface of Arctic during winter (right). Results without de-trending (Fig. 4a) and with de-trending (Fig. 4b) both are presented, which are seen very similar to both the Max and Min phases, respectively. Such a strong signature strengthens the idea of a solar influence from the top. This pattern of geopotential height is consistent with the annular ring structure of the NAM at the surface and upper stratosphere, as suggested by previous work^13^. Thus on average, the solar Min has a more negative NAM than solar Max and vice versa. Min minus Max years (Fig. 4a,b, bottom panel) suggest the strongest NAM signature (check the extended range of colour scale bar), which resembles a negative pattern. A significant rise in geopotential height of 36 m and 12 m is observed for 30 mb and 925 mb level, respectively, during solar Min years compared to that during Max years. Unlike air temperature, the results of the strong influence of the sun on Arctic geopotential heights are seen as practically unaffected by any trend related issues.

To verify whether similar polar vortex features, in the form of an annular ring structure, are constricted at different pressure levels within a cylindrical structure covering the upper stratosphere down to the lower stratosphere of the Arctic, we plotted results for several other pressure levels in Figs S2 and S3 (for Min years) and Fig. S4 (Max years). For Considering the annular structure in various arbitrary pressure levels, in the stratosphere (50 mb, 100 mb) as well as the troposphere (250 mb, 500 mb, 850 mb), opposite annular patterns are notable throughout these pressure levels for Min years compared to Max years. Results for various dynamic features include geopotential height (left) and zonal wind (right), suggesting consistency with air temperature (middle panel) and thus supporting the theory of downward propagation from a perturbed polar vortex^13^. For Max year, the data with or without de-trending suggest great similarity, and hence only de-trended version is presented (Fig. S4). For the Min year, there are little differences and hence both the version without de-trending (Fig. S2) and with de-trending (Fig. S3) are shown; however, the main pattern is found the same. Results are also consistent around the tropospheric level of a vertical column, as noted in Fig. 3.

In summary, for solar Min years, the warm air column is associated with positive geopotential height anomalies and an easterly wind, which reverses during Max years. Such NAM feature is clearly evident supporting the hypothesis of communicating a solar signal to Arctic via winter NAM.

To examine further, results of Eliassen-Palm (EP) flux^38^ is also presented (Fig. S5). It is a measure of planetary wave activity and its direction indicates the direction of wave propagation. The vertical components of the E-P flux are approximately proportional to the eddy heat, whereas horizontal components to momentum flux. Results of EP Flux of one solar Min (2010) and Max (1992) is presented for Feb (Fig. S5). Those (years/month) were chosen as representatives because their results matched with the observation of Min and Max phase in general for geopotential height, air temperature and zonal wind (as noted in Figs 3 and 4). In Fig. S5, solar Min (b) suggests an increase in wave driving around upper stratosphere in a horizontal direction to that from Max year (a). It causes a deceleration of winds which subsequently is responsible for the downward propagation of the anomalous easterly wind.

### Eliminating ENSO and similar signals

**Figure 5:**
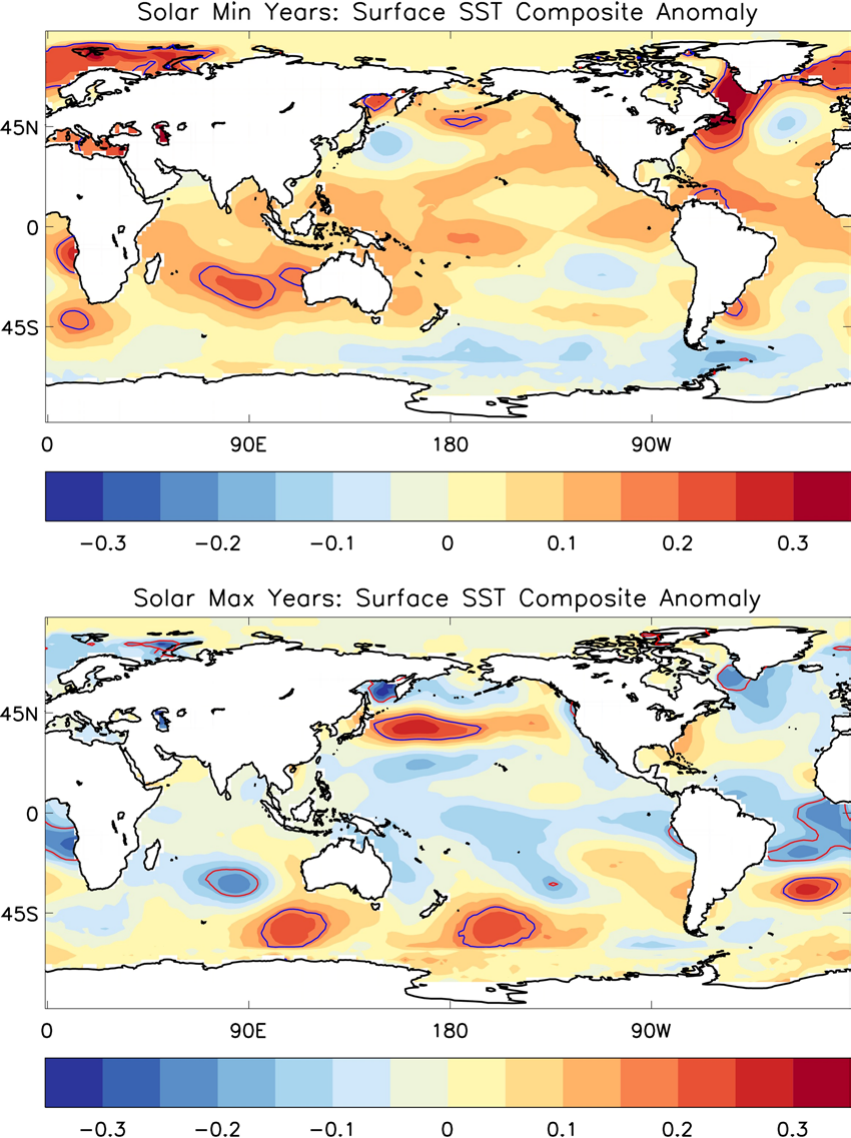
Tropical Pacific SST composites using NOAA Extended V4 (ERSST) data for solar Max (Top) and Min years (Bottom) during DJF. Levels usually significant up to 95% level are overlaid by opposite coloured contour. Plots are generated using IDL software, version 8 with the data from NOAA/OAR/ESRL PSD, Boulder, Colorado, USA, from their website at (http://www.esrl.noaa.gov/psd/).

Previous studies have suggested an influence of ENSO on the winter polar vortex^16,17^ and Arctic surface climate^22^. To examine the influence of ENSO on the above analysis, we analysed sea surface temperatures (SSTs) during solar Min and Max years (Fig. 5). Results show that SST anomaly along the central tropical Pacific (Nino 3/3.4 region) is nominal in either of the two solar phases. Interestingly, substantial warming/cooling around Arctic for top/bottom panel is clearly detected. It indicates, ENSO is not influencing the detected solar signal around Arctic and is cancelled out when the whole 38 years period is considered. It is also verified by Table 2, where it is noticed that equal number of ENSO positive and negative phase occurred during Min years (11 each), and also in Max years (8 each).

**Table 2:** ENSO and QBO phases are marked for years used in Solar Max and Min compositing.

Solar Cycle No	ENSO				QBO			
	(Solar Min)		(Solar Max)		(Solar Min)		(Solar Max)	
	+ve	−ve	+ve	−ve	W-ly	E-ly	W-ly	E-ly
21		1984	1979	1981	1986	1984	1979	1980
		1985	1980	1982		1985	1981	1982
		1986	1983				1983	
22	1987	1996	1990	1989	1988	1987	1991	1989
	1988		1991		1995	1994	1993	1990
	1994		1992			1996		1992
	1995		1993					
23	1998	1997	2003	1999	1998	1997	1999	2001
	2004	2006		2000	2005	2004	2000	2003
	2005			2001	2007	2006	2002	
	2007			2002				
24	2010	2008		2014	2009	2008	2014	
	2015	2009			2011	2010		
	2016	2011			2016	2012		
		2012				2013		
		2013				2015		
Total	11	11	8	8	9	13	9	7

Similar like the inter-annual variability of ENSO, QBO which is quasi-biannual in nature (follows nearly 28 months cycle) also has shorter time scale variability. It might have an influence on year to year variability of polar vortex features^18^ and subsequently to Arctic surface climate, as discussed earlier. However, in the current form of compositing analysis, it is believed that a near regular occurrence of both QBO phases is very likely during two different solar phases. It is also observed in Table 2 that the difference between W-ly and E-ly phase of QBO in Min or Max years are not considerably higher. Hence QBO signal (like ENSO) around Arctic may also be eliminated from the detected solar signature. Using Table 2, it is also possible to check whether the combined influence of SSN and QBO affected the results of detected solar signal or not. As we used QBO height 30 hPa, following previous discussions^18,20,21^, solar Min/Wly will always have a cold polar upper stratospheric temperature; whereas, for solar Max either in Wly or Ely phase, both warm, it will not affect our results of solar signal. A recent study showed that PDO could influence Arctic climate^23^. As no distinct PDO phase is noticed (weak SST signature around Aleutian Low) in plots of Fig. 5, it suggests that PDO cannot make much influence on the detected Arctic signal.

All those discussions indicate more on the robustness of identified solar signal around Arctic climate (on SST, air temperature etc.) following the methodology used.

### Influence from previous Season and years

During winter, the influence from the past season (SON) is evident on the surface and atmospheric temperatures, as years with low autumn ice cover tend to have pronounced warming in winter^39,40^ which, in turn, slows ice growth^41^. It is evident in Figure S6(a), which shows a strong connection between winter (DJF) and autumn (SON) total sea ice extent on a year to year basis. Here we are mainly interested about absolute sea ice extent. Demarcating dotted lines show when there is a positive (negative) anomaly on SON sea ice extent; it is usually associated with a positive (negative) sea ice anomaly of corresponding DJF of the subsequent year. The main purpose of this scatter plot (Fig. S6 (a)) is to show that the contribution from the previous season/year may have an influence on the subsequent winter sea ice level on a year to year basis, which is already noted in many previous studies^39–41^.

Polar amplification as a result of sea ice loss from previous seasons and years could explain the rapid declining trend of sea ice in recent years (Fig. 1a, bottom). Instead of 5 Min years, as observed for earlier cycles (one solar max to the next), it continued with 10 Min years in a succession (2004–2013), for last decade (Fig. 1a, top). It could thus accumulate more solar warming on a cumulative scale. As influence from previous seasons is one crucial factor^39–41^, which is subsequently also related to the previous year, 2017 winter even experienced more sea ice loss than from 2016 and experienced the maximum decline for the entire period of analysis. In the last weak solar cycle, there was only one year (2014) that exceeded the threshold of SSN 100. In contrast, all previous solar cycles (Fig. 1a, top) comprised at most 5 solar Max years. There was an uneven distribution of Max and Min years since 2004 (10 Min-1 Max-2 Min), in contrast to previous years (5 Max-5 Min). It is noteworthy that the trend in air temperature (Fig. 1b, top) around Arctic (which is warming) and Eurasian sector (cooling) suggests completely opposite to results of compositing during solar Max years (Fig. 2, Top). Moreover, in Fig. 2 for solar Min also suggests similarity even if the analysis is restricted only up to 2009 (not shown here). It is also in agreement with the results of the trend as noted in Fig. 1b (top). Our result thus suggests the latest rapid decline of Sea ice around Arctic in the recent decade and associated features^27–29^ could also have, contributions from the current weaker solar cycle. It had only one solar Max year, preceded by 10 Min years and thus cumulative influence from rest solar Min years could also have played a role in the current decline of 2017.

In Fig. S6(a), we mainly focused on the feature of ‘year to year variability’ of overall sea ice and did not discuss specifically about solar max or min years. To examine that feature of year to year variability further, regionally, we plotted Fig S6(b). Though a trend line is noticed in Fig. S6(a) but here we show it might not be distinct in all places when focus is on specific regions ‘A’, ‘B’ and ‘A + B’. The location of the Barents sea area (region A) suggests more spread in terms of sea ice extent for both the seasons of SON and DJF. Whereas, region B indicates lesser variability. Similar to Fig. S6(a), a trend is also noticed for region A and A + B, though not very distinct for ‘B’ (also check the scale). The purpose of chosing region A + B is to show that inspite of differences in region A and B, the overall sea ice trend is dominated by region A, which again resembles region A + B. However, the lesser variability of region B is also captured in A + B and hence A + B shows a closer fit for the overall sea ice extent to that from the specific region of either A or B. Solar max and min are distributed all over places in all three regions without showing much biases.

Fig. S7 depicts time series plot in those three regions for a) sea ice extent and b) air temperature at 1000 mb. In both cases, a strong trend is noticed for region A, which is the least for region B (check the scaling of the y axis). As expected, region A + B suggests a moderate trend. In terms of trend, the sea ice extent and air temperature show consistency in respective regions, as expected^28^. The reduced trend in region B may explain why the air temperature plot for solar signature (Fig. 2) suggests largest signal from the other regions, after detrending the data. Whereas, due to a stronger trend in region A, the solar signal seems substantially reduced after detrending. The stronger trend in region A is also consistent with previous studies^34,36^. Following the observation from Figs S6 and S7 relating to a least trend in the region B, and noting a solar-related air temperature anomaly in Fig. 2, it can be hypothesized that some of the reduced winter sea ice in recent years, may in part, be a result of the current weaker solar cycle, where region B suggests a stronger influence. Moreover, following Fig. 2, it can be stated that the accumulation of more snow around Eurasian sector in recent winters may also have a contribution from the current weaker solar cycle.

To eliminate trend and other issues related to the nonlinear coupling that might affect results of compositing, we additionally applied the method of Multiple Linear Regression Technique (MLR), which is another robust technique often used in detecting solar influence.

### Results of Regression and Correlation

**Figure 6:**
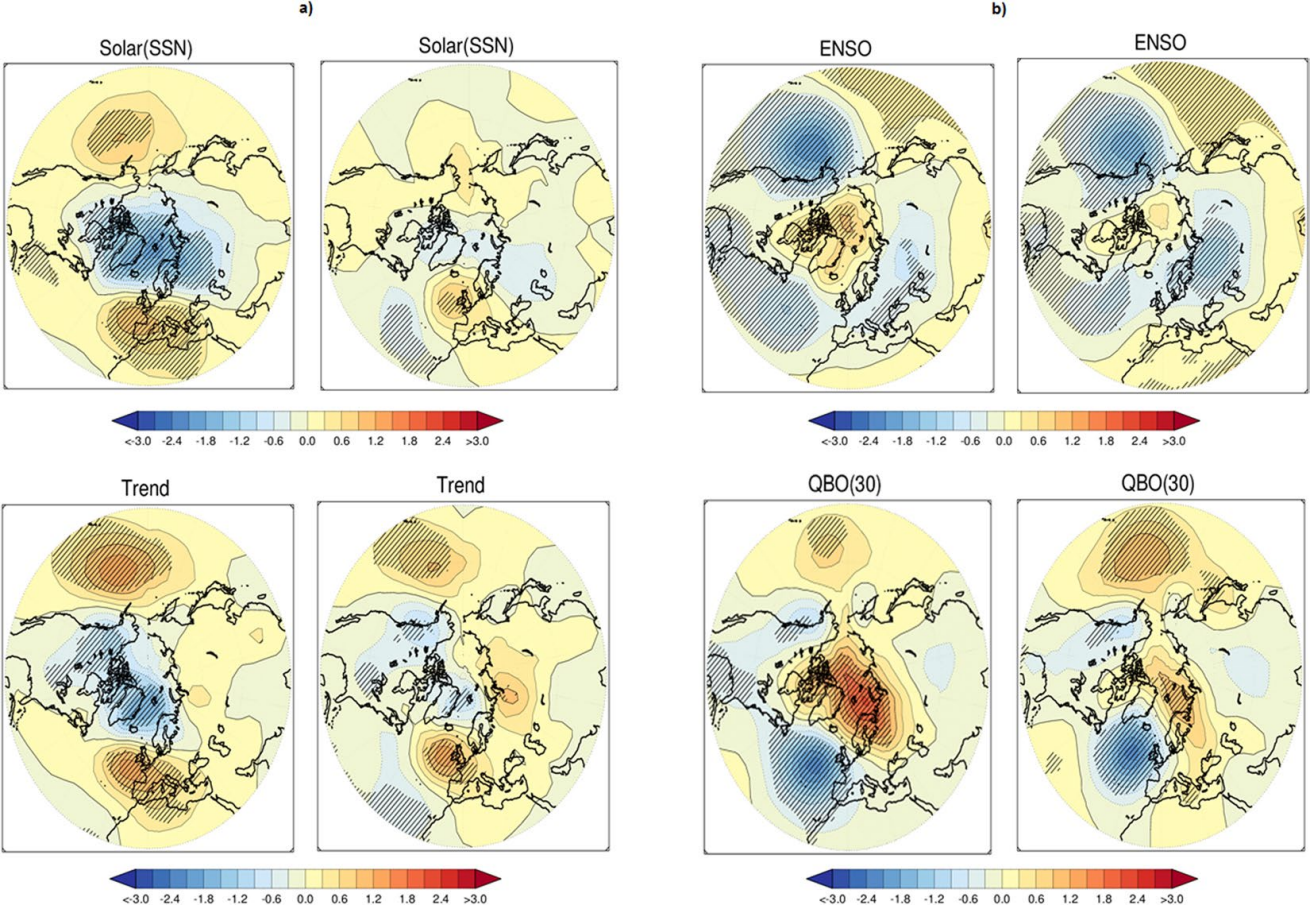
The signal (Max–Min, hPa) in DJF, Hadley centre SLP data, obtained from a multiple linear regression (MLR) analysis over the 1979–2012 period. Independent indices used are the trend, SSN, ENSO, AOD (volcano), QBO (30hPa) and AO. Results without using AO as one index in the MLR are presented in the left, and with AO in the right. Signals are shown for (**a**) SSN and trend; (**b**) ENSO and QBO. Negative contours are shown by dotted lines. Shaded regions are estimated significant at the 95% level using a two-sided Student’s t-test. Note here the results of (Max-Min) are presented. Plots are prepared using IDL software, version 8.

Arguments may be raised whether the solar signal - as computed using compositing method - could be an artefact of the methodology used. To strengthen the findings further, the MLR technique with AR (1) noise model is applied. Here it is possible to segregate the influence of linear trend. Other strong individual signals from QBO and ENSO can also be eliminated. To be compatible with compositing technique, MLR is also applied with the trend as one regressor (Fig. 6) and also without trend (Fig. S8). Figure 6a (top) presents signature from the sun (note here that it is Max-Min), after removing the trend, volcano, ENSO and QBO (left). In addition to those, AO is also included as one regressor, in the right. To have consistency with geopotential height, MLR is applied on Sea Level Pressure (SLP) and the data used is from Hadley Centre. A clear annular ring structure around the Arctic is very distinct (Fig. 6a, left), which is again consistent with the solar signature as detected using compositing. Interestingly, the annular ring structure completely disappears in the right, indicating a pathway involving AO. It is noteworthy that solar signature around the Arctic in other seasons is not detected using the MLR technique. In the compositing technique also, the solar signal in rest seasons (other than DJF, shown in Fig. S1), was found to have reduced its strength when the analyses were done after de-trending the data (not shown here). Hence, both the study MLR, as well as compositing, suggests consistency in terms of solar signal.

The role of trends in the Arctic is well documented^3,4,27^. Previous studies even suggested the role of ENSO not only on Arctic surface climate^22^ but also on the winter polar vortex^16,17^. The same is also true for QBO^42,43^. Similar MLR analysis was applied to detect an independent signature of the trend (Fig. 6a, bottom), ENSO and also for QBO (Fig. 6b). It confirmed that Arctic climate is indeed strongly influenced by their variability. The strongest influence is detected due to the sun and QBO, which is followed by the trend and ENSO. To further highlight a stronger influence of the sun than that from the trend on SLP, Fig. S8a presents the result of regression for ‘SSN-Trend’, using outputs of Fig. 6a (left). It indicates a larger influence of the sun than the trend around the Arctic and the difference is stronger in locations of region A. Even excluding the strong influence of the trend and also the sun, the individual influence for ENSO and QBO around Arctic is still distinct (Fig. 6b, left). The pathways to influence Arctic via AO as discussed earlier is also noticed (right). If AO is included in the regression, the significant region around the Arctic completely disappears for ENSO and reduces considerably for QBO. The strongest reduction of signal due to AO is noticed for the sun. Using MLR, the significant signal for ENSO and QBO around the Arctic in other seasons is not detected. When the MLR analysis is applied without considering the trend as a regressor (Fig. S8b), the signal for the sun, ENSO and QBO suggest similarity, as observed in Fig. 6. It is again consistent with the results of compositing (Fig. 4a,b).

All the above analyses indicate that solar cyclic signature on winter Arctic climate may not simply be an artefact of the particular methodology used. Every technique has its inherent limitations and strengths, but a similar solar signature is identified around Arctic climate, even using different techniques. The compositing technique though arbitrarily focused on SSN threshold above and below 1.35 (SSN 100), using the mean value of SSN, the results are also unaltered.

**Figure 7:**
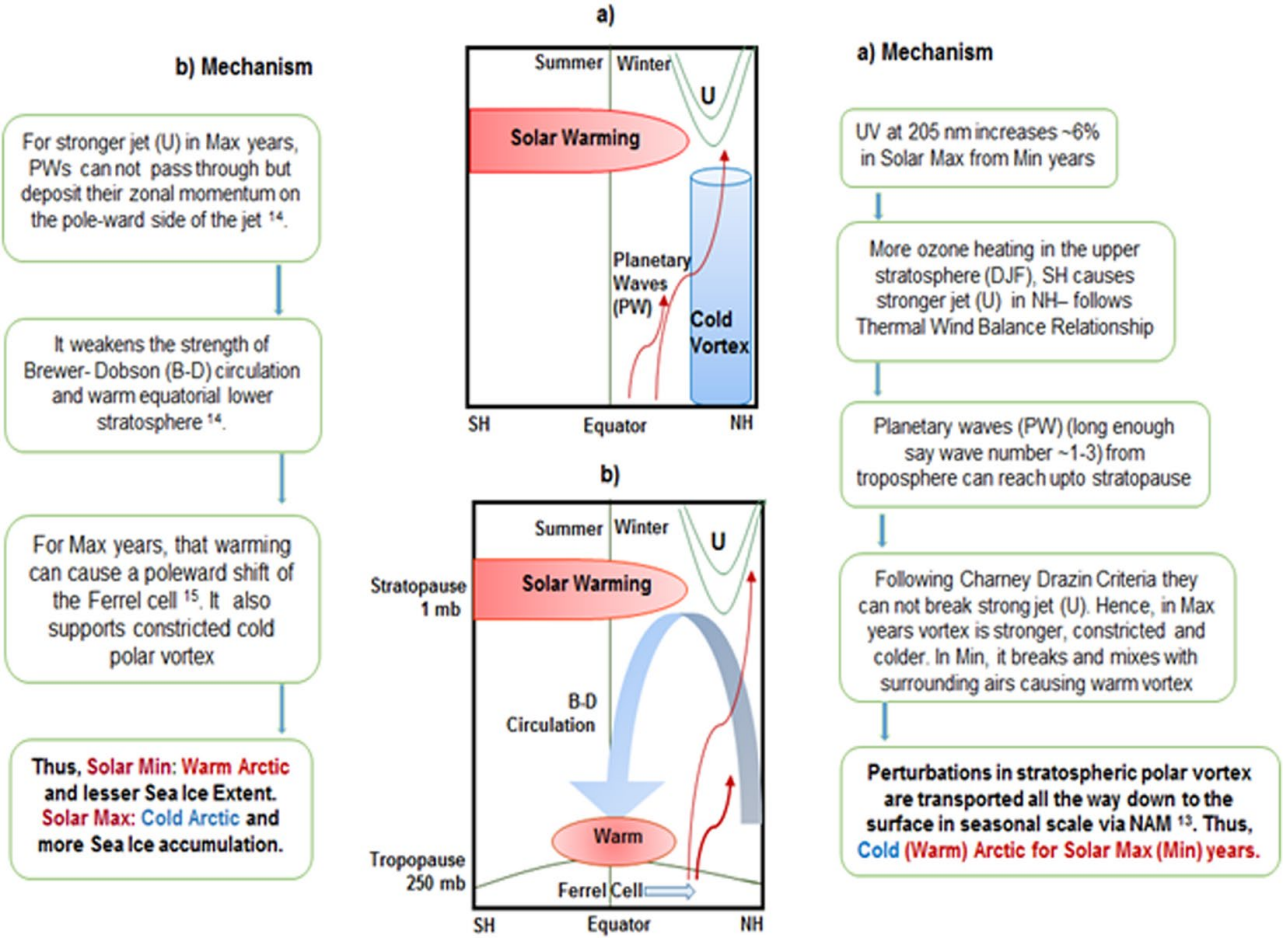
Mechanism to describe the stratospheric pathway for solar cycle variability to influence the Arctic climate. Mechanisms for (**a**) discuss a route where perturbation in the upper stratospheric polar vortex is transported downwards and impacts the Arctic on a seasonal scale via the winter NAM (flowchart is presented on the right). Mechanisms for (**b**) discusses the route that involves upper stratospheric polar vortex, tropical lower stratosphere, Brewer-Dobson circulation and Ferrel cell (flowchart is presented to the left). It is created using images or clip art available from Powerpoint.

Finally, we present a diagram and flowchart (Fig. 7) to illustrate the mechanisms of solar cycle variability and its influence on the Arctic climate through stratospheric pathways. The route (a) where perturbation in the upper stratospheric polar vortex is transported downwards and modulates the Arctic in a seasonal scale via the winter NAM is discussed with the help of a supporting schematic. Another route (b) involving upper stratospheric polar vortex, tropical lower stratosphere, Brewer-Dobson circulation and Ferrel cell is also presented. Though our results did not involve that route (b), the solar influence on the Arctic via Ferrel cell is found to agree with previous observations and reinforces the findings ‘Solar Max (Min) - cold (warm) Arctic.

To further extend our understanding, we focused on Arctic sea ice extent and applied a simple correlation technique. In this technique, only two variables are considered, while nonlinear coupling amongst parameters is considered immaterial. In spite of the presence of possible coupling (e.g., a nonlinear coupling existing between the sun and ENSO)^24–26^, correlation can identify a probable connection between two parameters, provided a mechanism exists in support. Such knowledge could be useful for prediction purposes as separating out individual signal could sometimes be challenging or unrealistic.

**Figure 8:**
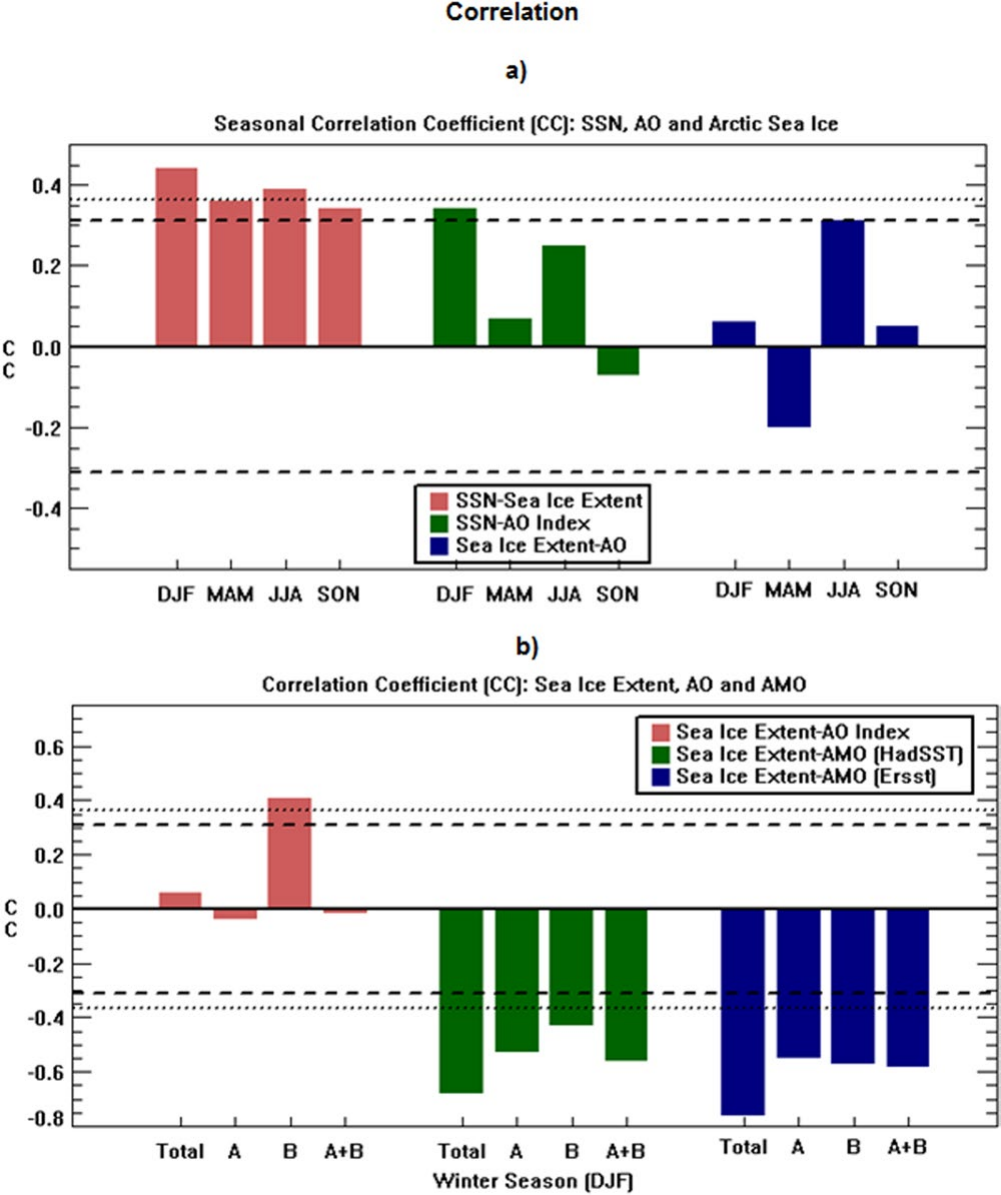
Results of Correlation Coefficient (c.c) between Sea Ice Extent and various other parameters. (**a**) Seasonal c.c. for four different seasons are presented using other parameters as SSN and AO, and (**b**) c.c. for the winter season in different regions using other parameters as AO and AMO. Significant levels of 95% and 99% using a students ‘t-test’ are marked by dashed line and dotted line respectively. Plots are prepared using IDL software, version 8.

During DJF, Arctic sea ice extent suggests a strong correlation with SSN (99% significant) and even with AOD (95% significant) (Table 3a). SSN is also found to be strongly correlated with AO (95% significant). Figure 8a shows that significant correlation between Arctic sea ice extent and SSN is still present in other seasons as well. However, the correlation between SSN and AO is only significant in DJF, confirming that the possible route of solar influence on winter Arctic sea ice is via the AO. On the other hand, the influence of AO on Arctic sea ice extent is not present during winter. It is strongest during JJA, though fails to exceed a significant threshold of 95% level.

**Table 3:** Results of Correlation technique for various indices during DJF with the significant level at 95% and 99% are shown by bold and bold-italics respectively.

**a)**					
Correlation Technique	Correlation coefficient				
	SSN	ENSO	QBO	AOD	AO
Arctic Sea Ice Extent (DJF)	***0.44***	0.11	0.12	**0.33**	0.06
AO Index (DJF)	**0.34**	0.16	0.12	0.24	
**b)**					
**Correlation Technique**	**Correlation coefficient: AO and Arctic Sea Ice Extent (DJF)**				
	**Total Sea Ice Extent**	**Region A**	**Region B**	**Region A + B**	
Befor detrending	0.06	−0.04	***0.41***	−0.015	
After detrending	0.19	−0.06	***0.48***	−0.015

To explore further why the influence of winter AO is not present on overall sea-ice, we examined it regionally (Fig. 8b). Interestingly, we notice a strong significant correlation between AO and sea ice extent, which is only present for region B. That signal (99% significant) is still present even after removing the trend (Table 3b). However, the signal is missing for region B and A + B, which is similar to overall sea ice extent. Our study thus suggests, in spite of the presence of longer-term oceanic variability and other oceanic influences, it is still possible to detect a strong influence of the AO on winter sea ice around region B. The signature of AO is similar as we identified in the atmospheric column using other robust techniques. For sea ice, as ocean is likely to play the major role, AO signal might be masked by oceanic influences in other regions. The solar signal on correlation as observed in Fig. 8a disappears when the data is detrended, suggests regional sea ice can influence the result (as we already showed here). It could also be related to longer-term ocean variability.

To understand more about oceanic longer-term influence, those might affect/overtake results of solar cyclic fluctuations on sea ice extent, when data detrended, we analysed the role of winter Atlantic Multidecadal Oscillation (AMO). Many current studies^34,36^ indicated that the trend observed in Arctic sea ice in recent decades has primarily been caused by enhanced oceanic heat transport in North Atlantic. Oceanic variability is believed to have a significant impact on Arctic sea ice extent, among which major sources of natural variability are Atlantic meridional overturning circulation (AMOC)^44^ and AMO^45,46^. AMOC plays an important role in oceanic heat transport around the north Atlantic and both the AMOC and AMO indices are found significantly anti-correlated with Arctic sea ice extent^45,46^. It is suggested that the variability of AMO is driven by AMOC and an in-phase relationship exists in their observed cycles^44,47^. In terms of oceanic longer-term variability, here we particularly focus on the AMO and find a strong connection between sea ice and AMO in winter, agreeing with previous studies^45,46^. Earlier discussions suggested that there are few differences in region A and B relating to trend (Figs S6 and S7), but correlation technique indicated a very strong anti-correlation between the winter AMO index and sea ice in all regions of our considerations (Fig. 8b)). Even using two different data sources (HadSST and ERSST) we arrive at similar results, and it is also true for overall sea ice extent. It could also be possible that, in region B, due to a strong presence of AO influence of the sun, it may mask some of the influence of the longer-term trend (seen in Fig. 2) to suggest a lesser trend, as also noted in Figs S6 and S7.

## Discussion

Using the observational sea ice record and NCEP/NCAR atmospheric reanalysis data, we examined the role of the eleven-year solar cycle in the winter Arctic atmosphere and the sea ice cover. Solar Maximum and Minimum years are identified when the sunspot numbers are more or less than 1.35 standard deviations (i.e., SSN threshold 100 and around the mean). Composite analysis of solar Max and Min years reveals Arctic warming (cooling) during Min (Max) years that extends from the surface to the upper stratosphere.

This distinct annular ring structure throughout the atmospheric column suggests downward propagation of perturbed atmospheric behaviour from the upper stratospheric polar vortex. Associated dynamical features for geopotential height and zonal wind linked with specific temperature anomaly are also captured in two different phases of solar years. For solar Min years, there is an easterly zonal wind anomaly and positive geopotential height deviation, within the columnar profile, which is consistent with the constricted warm air. For Max years, a reversal of patterns occurs. De-trended data also suggests similarity. A significant solar influence is also detected on Eurasian air temperature, which is practically unaffected after detrending the data. In Eurasian sector, it however suggests a warming during Max years while cooling in Min. The detected solar signature was shown to be independent of ENSO. It also reduced the influence of other probable contaminating factors, e.g., QBO and PDO. Thus, this study indicated the robustness of detected solar signal, following the methodology of compositing.

This study indicates that the Arctic climate could, however, be different in any particular year, without following the rule as discussed. It could be due to local influences or the phase of ENSO and QBO, etc., whichever may dominate. But in spite of the presence of other signals and that year to year variability, the composite technique was still able to detect a strong influence during winter of the overall period of analysis that is due to the sun. Such analysis is no doubt, useful for improved future prediction.

To strengthen our findings further, the MLR technique is applied, where it is also possible to segregate the influence of linear trend, ENSO, QBO and volcano. A distinct solar signature around the Arctic, like an annular ring structure, on SLP is identified, which is consistent with earlier results as detected using compositing. Interestingly, that solar signal completely disappears if AO is included in the regression, indicating a pathway involving AO. Similar analyses to detect an independent signature of the ENSO and QBO also confirmed that Arctic climate is indeed strongly influenced by their variability, even excluding strong influences of the linear trend. The pathways of those effects emanate via AO. In MLR, the strongest influence around Arctic is detected due to the sun and QBO, which is followed by the trend and ENSO. During other seasons, those signatures neither from the sun, ENSO nor QBO around the Arctic are detected using the MLR technique. The main findings from compositing and MLR seem to be similar with or without trend. The whole study compared and contrasted two different techniques, Compositing and Multiple Linear Regression, but the main finding is the same; that the sun can modulate winter Arctic climate via the AO.

Our result suggests the latest rapid decline of sea ice around the Arctic in the recent winter decade/season could also have contributions from the current weaker solar cycle. The last 14 years are dominated by solar Min years and have only one Max. This is unlike other previous years, where the number of Max and Min years were evenly distributed (five each). The cumulative effect from the past 13 solar Min years could have played a role in the current record decline of the last winter, 2017. The current weaker solar cycle may also have contributions on increase in winter snow cover around the Eurasian sector.

Presenting schematics and flowcharts, we discussed mechanisms of how solar cycle variability influences Arctic climate. In the first route, perturbation in the upper stratospheric polar vortex is transported downwards and modulates the Arctic in a seasonal scale via the winter NAM. Another route was shown, which could involve upper stratospheric polar vortex, tropical lower stratosphere, Brewer-Dobson circulation and Ferrel cell. It could also reinforce the findings of the ‘Solar Max (Min) - cold (warm) Arctic’ scenario.

The association between solar variability and Arctic climate was further explored using Arctic sea ice extent, which is likely to be dominated by oceanic parameters. It indicated that the location of the Barents Sea has a major bearing on overall Arctic sea ice decline. We also applied the correlation technique and showed the solar influence via AO can even strongly modulate sea ice extent in the region covering the Labrador Sea and Greenland. In terms of longer-term oceanic influence, sea-ice extent (in a regional scale and also as a whole) suggests strong anticorrelations with the AMO.

## Methods

In this study, we rely on compositing techniques on various atmospheric and surface parameters, those include air temperature, geopotential height, zonal wind and sea surface temperature. Anomalies are computed relative to a base period of 1981–2010. The level of significance is determined by the method of mean differences and applying ‘t’ test. The data of Arctic sea ice extent is also explored. The period of analysis is from 1979 to 2016 inclusive. With the advent of satellite data, that period is more reliable, and also it covers more than 30 years (the criteria for a climatological period). Arctic climate also suggested an abrupt variation since 1979^1^ and hence the starting period of analyses is considered here from 1979.

We segregated the whole SSN range in two sets, above and below about standard deviation 1.35. Solar maximum (Max) years or High SSN are defined when SSN is more than 100 (SSN > 100) and Solar minimum (Min) years or Low SSN when it is less than that (SSN < 100). SSN value 100 is ~1.35 standard deviation. The years for Max and Min are presented in Table 1. As there is no observation of SSN between 95 and 100, using mean value (SSN = 95) as a threshold also suggests similarity. For the period under consideration (1979–2016), there is a total of 16 solar Max years and 22 solar Min years.

We also applied Multiple Linear Regression (MLR) technique, with AR (1) noise model, on DJF Arctic sea ice. Other independent factors considered are the trend, ENSO, QBO (30hPa), AO and Aerosol Optical Depth (AOD, to represent volcano). PDO being multi-decadal in nature was not used in the regression of 38 years analysis. Such methodology of MLR with noise model (order one) was extensively used in various climate studies^24,29–31,48^ among others. The code was developed by Prof Myles Allen, Oxford University (Personal Communication). In this technique, noise coefficients are estimated simultaneously with the components of variability and done so that the residual is consistent with an order one red noise model. It minimises the possibility of noise being interpreted as a signal. Finally, the level of confidence is determined using the Student’s t-test. Additionally, the technique of correlation is also applied where the significance is estimated using Student’s t-test.

The data for air temperature, geopotential height and zonal wind analysed is from NCEP/NCAR reanalysis product^49^. It is also available at http://www.esrl.noaa.gov/psd/.

For Sea Surface Temperature (SST), we used NOAA extended SST v4 (ERSST) data^50^. It is available from NOAA, Boulder, from their Web site at http://www.esrl.noaa.gov/psd/ and also from https://www.ncdc.noaa.gov/data-access/marineocean-data/extended-reconstructed-sea-surface-temperature-ersst-v4/.

For SLP, the in-filled HadSLP2 dataset^51^ and available from 1850 to 2004 are used. It can also be found from http://www.metoffice.gov.uk/hadobs/hadslp2. It has been updated up to 2012 using HadSLP2r_lowvar data^52^ (http://www.metoffice.gov.uk/hadobs/hadslp2/data/download.html).

Data of Arctic sea ice extent is provided from the National Snow and Ice Data Centre (NSIDC) [http://nsidc.org/data/seaice_index/]. This data set provides daily and monthly summaries of total sea ice extent, defined as the sum of all areas covered by at least 15% sea ice concentration, using the NASA Team sea ice algorithm^53^. For regional sea ice extent the data of NSIDC is obtained from KNMI Climate Explorer (http://climexp.knmi.nl).

SSN is used to determine solar eleven-year cyclic variability (version 2), which is available from http://www.sidc.be/silso/versionarchive and described in literature^54^. SSN has a strong correlation with solar UV variability, visible solar irradiance and solar F10.7 (it is solar flux with bandwidth 10.7 cm (2800 MHz), which can be measured in the ground). Thus SSN can also serve as a proxy for those solar indices.

Various other indices used in this study (ENSO, AO, AMO and AOD) can be obtained from KNMI Climate Explorer (http://climexp.knmi.nl). Data are also available from following sites: (AO: http://www.cpc.ncep.noaa.gov/products/precip/CWlink/daily_ao_index/ao.shtml; AOD: http://data.giss.nasa.gov/modelforce/strataer/ENSO: http://www.cpc.noaa.gov/data/indices/). QBO at 30 hPa level is from the NCEP/NCAR reanalysis and obtained from http://www.cpc.ncep.noaa.gov/data/indices/qbo.u30.index. For AMO, two different data sources are used, one based on HadSST 3.1.1.0 data from UK Met Office and the other from ERSST v5 from National Climate Data Centre (NCDC), NOAA.

## Electronic supplementary material

Supplementary Information
