# Supplementary material for: Solar cyclic variability can modulate winter Arctic climate
Source: Sci Rep. 2018 Mar 20;8:4864. doi: 10.1038/s41598-018-22854-0 (PMC5861038; doi:10.1038/s41598-018-22854-0)
Supplement: Supplementary file 1 — Supplementary Information [file 41598_2018_22854_MOESM1_ESM.pdf]

Solar cyclic variability can modulate winter Arctic climate

Indrani Roy
University of Exeter
(I.Roy@exeter.ac.uk)

Supplementary Figures:

Fig. S1. The Composite anomaly of Air temperature (1000mb) at MAM, JJA, SON, and Nov during Solar Min years (a) and Solar Max years (b). Here values of annual average SSN is used. Significant regions up to 95% level are marked by the blue contour. Plots are prepared using Met Office IDL (MIDL) software and also verified with the plots generated by the NOAA/OAR/ESRL PSD, Boulder, Colorado, USA, from their website at (http://www.esrl.noaa.gov/psd/).

Fig. S2. Polar vortex annular features are captured for Arctic (DJF) from the stratosphere down to the lower troposphere during **Solar Min** years. Composite anomalies of Geopotential Height (left column), Air temperature (middle), and zonal wind (right) are shown at various arbitrary levels in the stratosphere (50mb, 100mb) and troposphere (250mb, 500mb, 850mb). Plots are prepared using Met Office IDL (MIDL) software and also verified with the plots generated by the NOAA/OAR/ESRL PSD, Boulder, Colorado, USA, from their website at (http://www.esrl.noaa. gov/psd/).

Fig. S3. Same as Fig S2 for **Solar Min**, but the data is de-trended before the analyses. Plots are prepared using Met Office IDL (MIDL) software.

Fig. S4. Same as Fig S2, but for **solar Max** and the data is de-trended before the analyses. Plots are prepared using Met Office IDL (MIDL) software.

Fig. S5. EP-Flux computed from temperature and the wind (daily-averaged values) obtained from the NCAR/NCEP Reanalysis. Results of one Solar Max 1992 (a) and one Min (2010) (b) for one winter month (February) is presented. Contours show the value of EP Flux convergence/divergence. Solid lines show areas of EP-Flux convergence, and hence of westerly deceleration. For display purposes, the EP-Flux vectors above 100mb are multiplied by a scale factor 5. Plots are generated by the NOAA/OAR/ESRL PSD, Boulder, Colorado, USA, from their website at (http://www.esrl.noaa. gov/psd/).

Fig.S6. Arctic Sea ice extent (million Sq-km) of solar Max and Min years, DJF against Sea ice extent of the previous season (SON). a) Anomaly plot for total sea ice extent; b) Sea ice extent for regions A, B and A+B respectively. Plots are generated using IDL software version 8.

Fig.S7. Time series plot for winter (DJF) Arctic sea ice extent (million Sq-km) (a), and mean air temperature (ºC) at 1000mb (b), in regions A, B and A+B. Plots are generated using IDL software version 8 (a) and Met Office IDL (MIDL) software (b).

Fig.S8. The signal (hPa) in DJF, Hadley centre SLP data, obtained from a multiple linear regression (MLR) analysis over the 1979–2012 period. The results of 'SSN-Trend' that used the output from Fig. 6a (left) is presented in a). For b) independent indices used are SSN, ENSO, AOD (volcano) and QBO (30hPa). It is similar as Fig. 6, but the trend is not considered here. Signals are presented for SSN, ENSO and QBO as shown by subtitles. Negative contours are shown by dotted lines. Shaded regions are estimated significant at the 95% level using a two-sided Student's t-test. Note here the results of (Max-Min) are presented. Plots are prepared using IDL software, version 8.

a) Solar Min Years

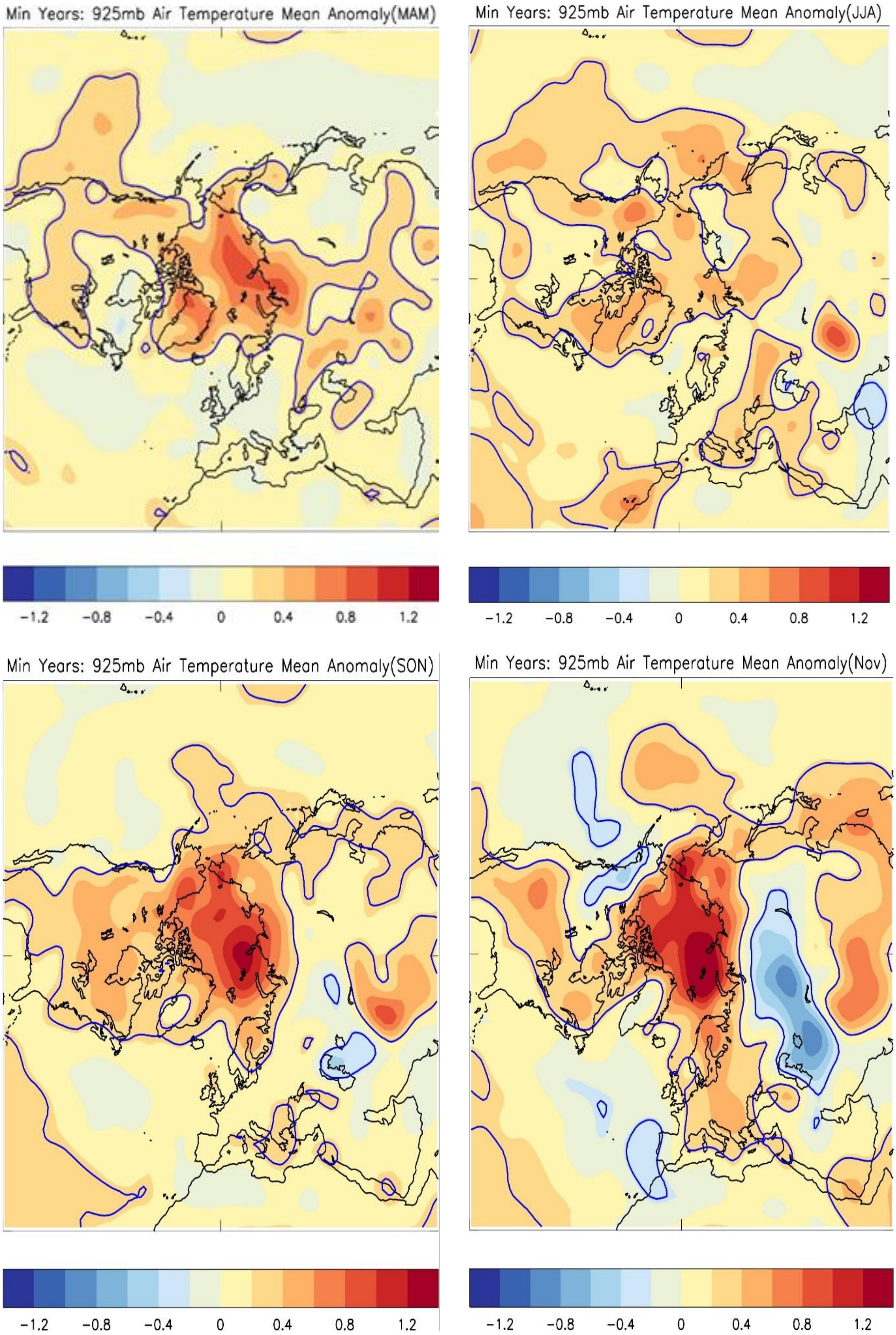

b) Solar Max Years

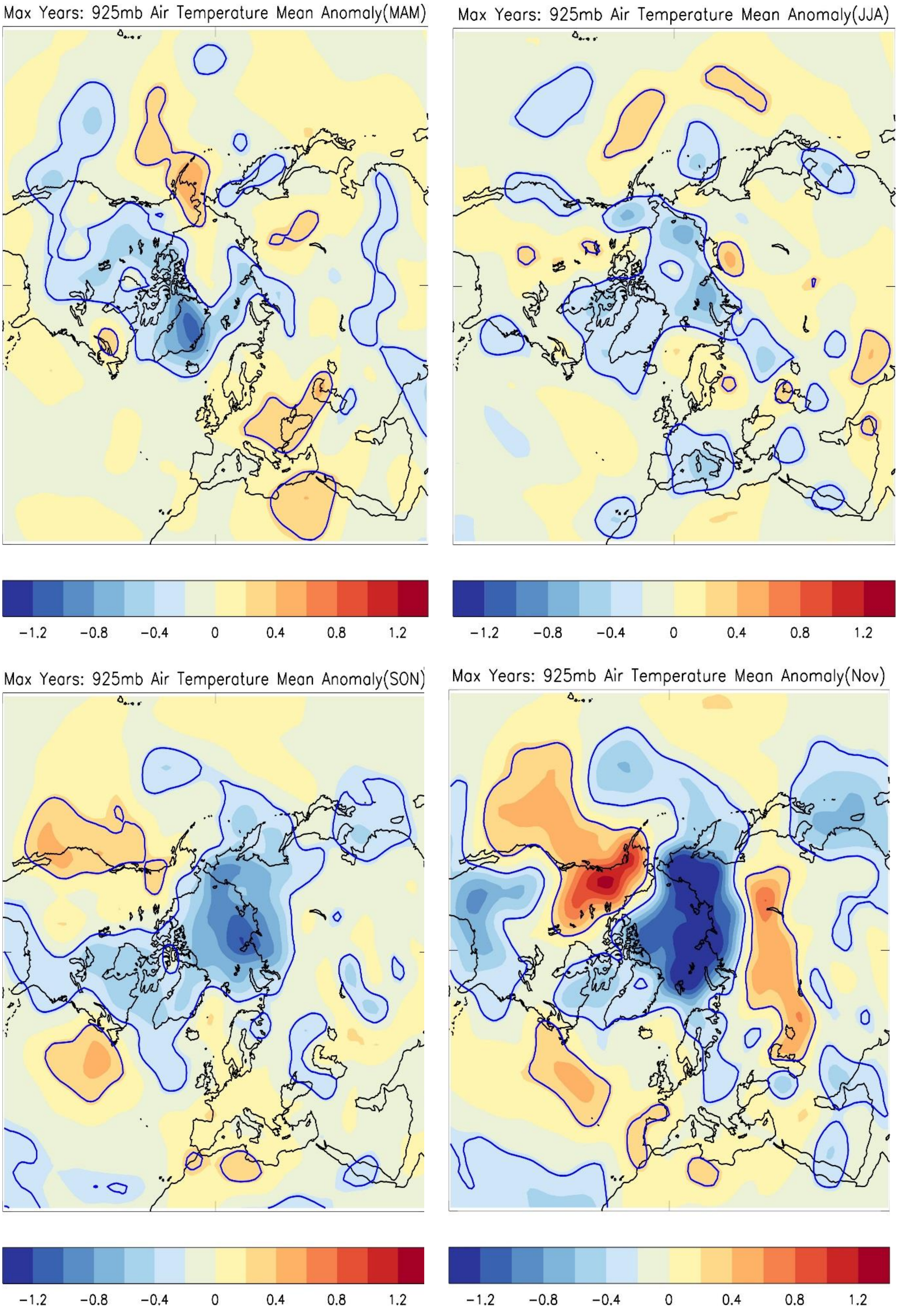

Fig. S1: The Composite anomaly of Air temperature (ºC) at 1000mb for MAM, JJA, SON, and Nov during Solar Min years (a) and Solar Max years (b). Here values of annual average SSN is used. Significant regions up to 95% level are marked by the blue contour. Plots are prepared using Met Office IDL (MIDL) software and also verified with the plots generated by the NOAA/OAR/ESRL PSD, Boulder, Colorado, USA, from their website at (http://www.esrl.noaa.gov/psd/).

Solar Min Years

Geopotential Height **Air Temperature** **Zonal Wind**

50mb

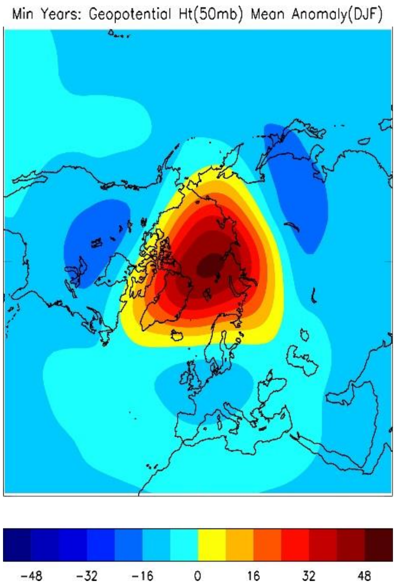

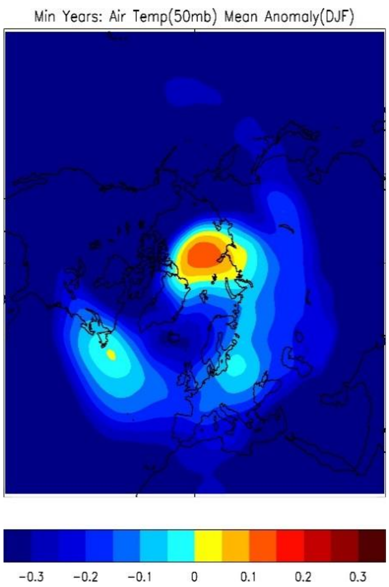

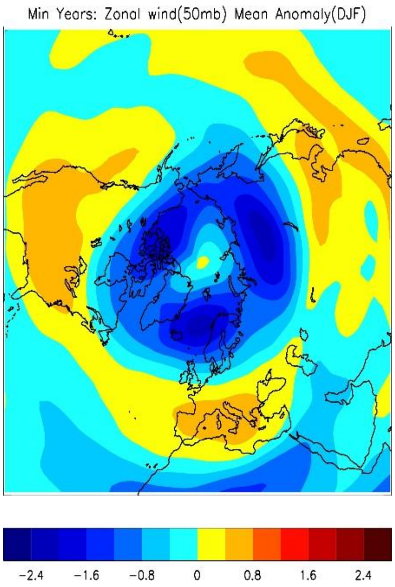

100mb

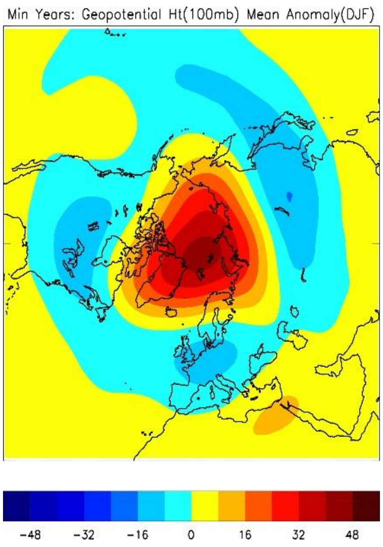

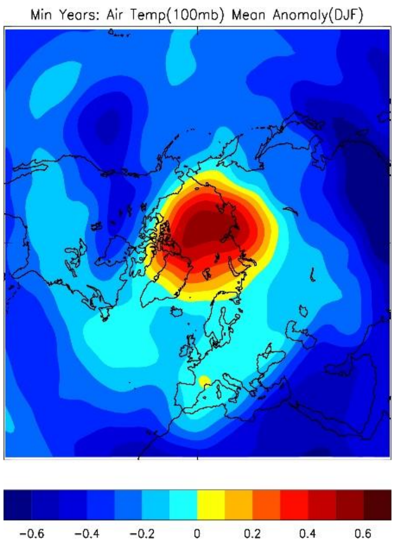

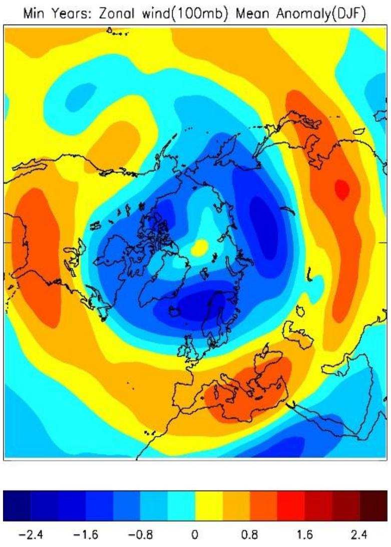

250mb

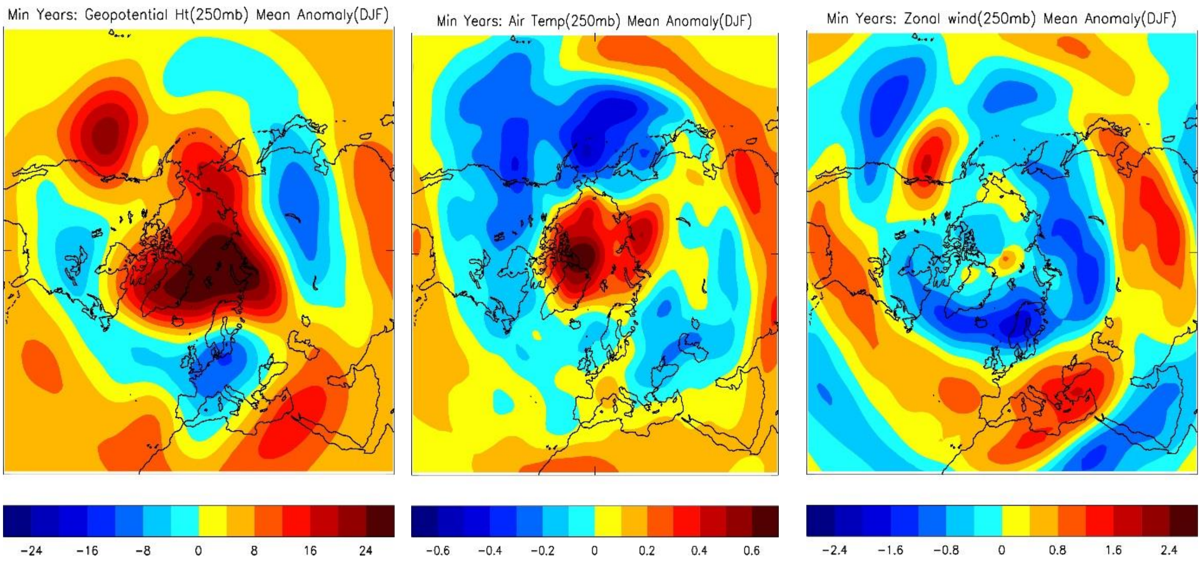
Min Years: Geopotential Ht(250mb) Mean Anomaly(DJF)
−24 −16 −8 0 8 16 24
Min Years: Air Temp(250mb) Mean Anomaly(DJF)
−0.6 −0.4 −0.2 0 0.2 0.4 0.6
Min Years: Zonal wind(250mb) Mean Anomaly(DJF)
−2.4 −1.6 −0.8 0 0.8 1.6 2.4

500mb

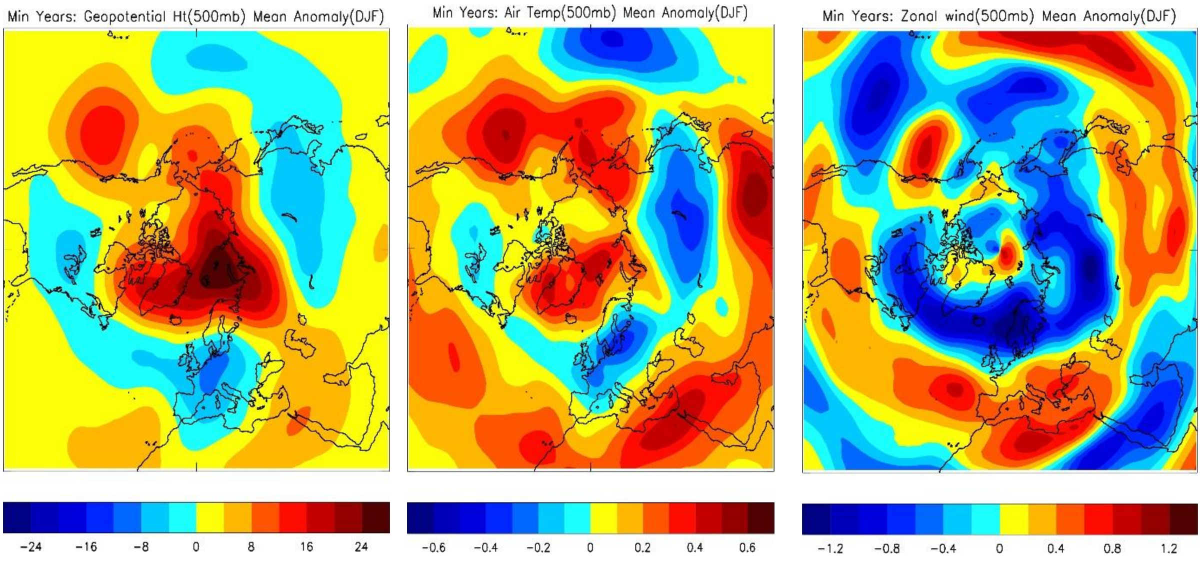
Min Years: Geopotential Ht(500mb) Mean Anomaly(DJF)
−24 −16 −8 0 8 16 24
Min Years: Air Temp(500mb) Mean Anomaly(DJF)
−0.6 −0.4 −0.2 0 0.2 0.4 0.6
Min Years: Zonal wind(500mb) Mean Anomaly(DJF)
−1.2 −0.8 −0.4 0 0.4 0.8 1.2

850mb

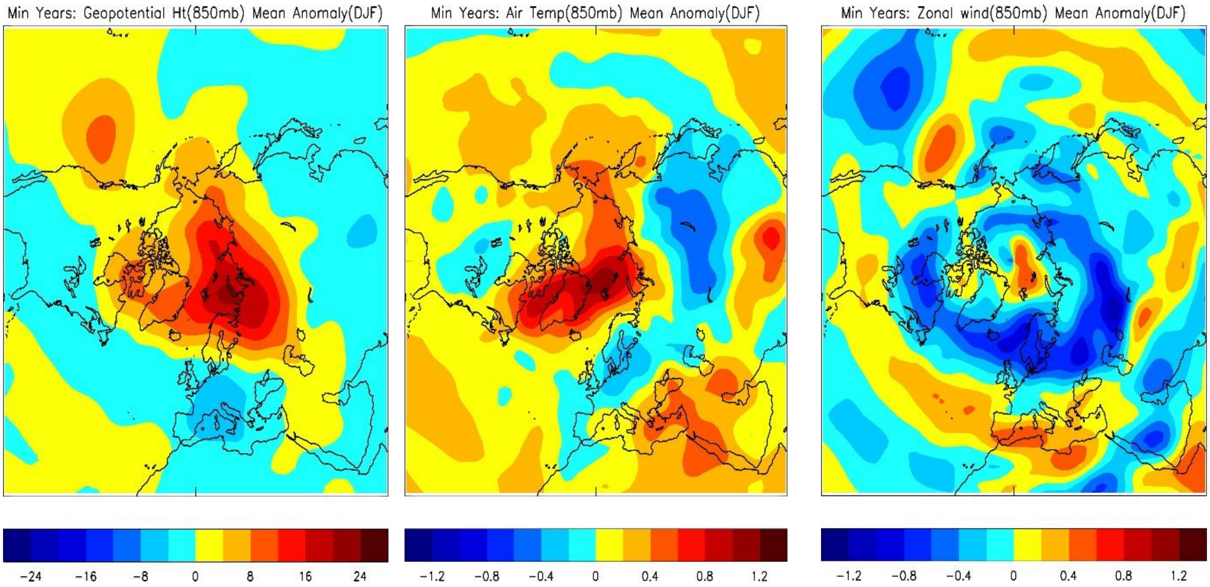

Fig. S2. Polar vortex annular features are captured for Arctic (DJF) from the stratosphere down to the lower troposphere during **Solar Min** years. Composite anomalies of Geopotential Height (m) [left column], Air temperature (ºC) [middle], and zonal wind (m/s) [right] are shown at various arbitrary levels in the stratosphere (50mb, 100mb) and troposphere (250mb, 500mb, 850mb). Plots are prepared using Met Office IDL (MIDL) software and also verified with the plots generated by the NOAA/OAR/ESRL PSD, Boulder, Colorado, USA, from their website at (http://www.esrl.noaa.gov/psd/).

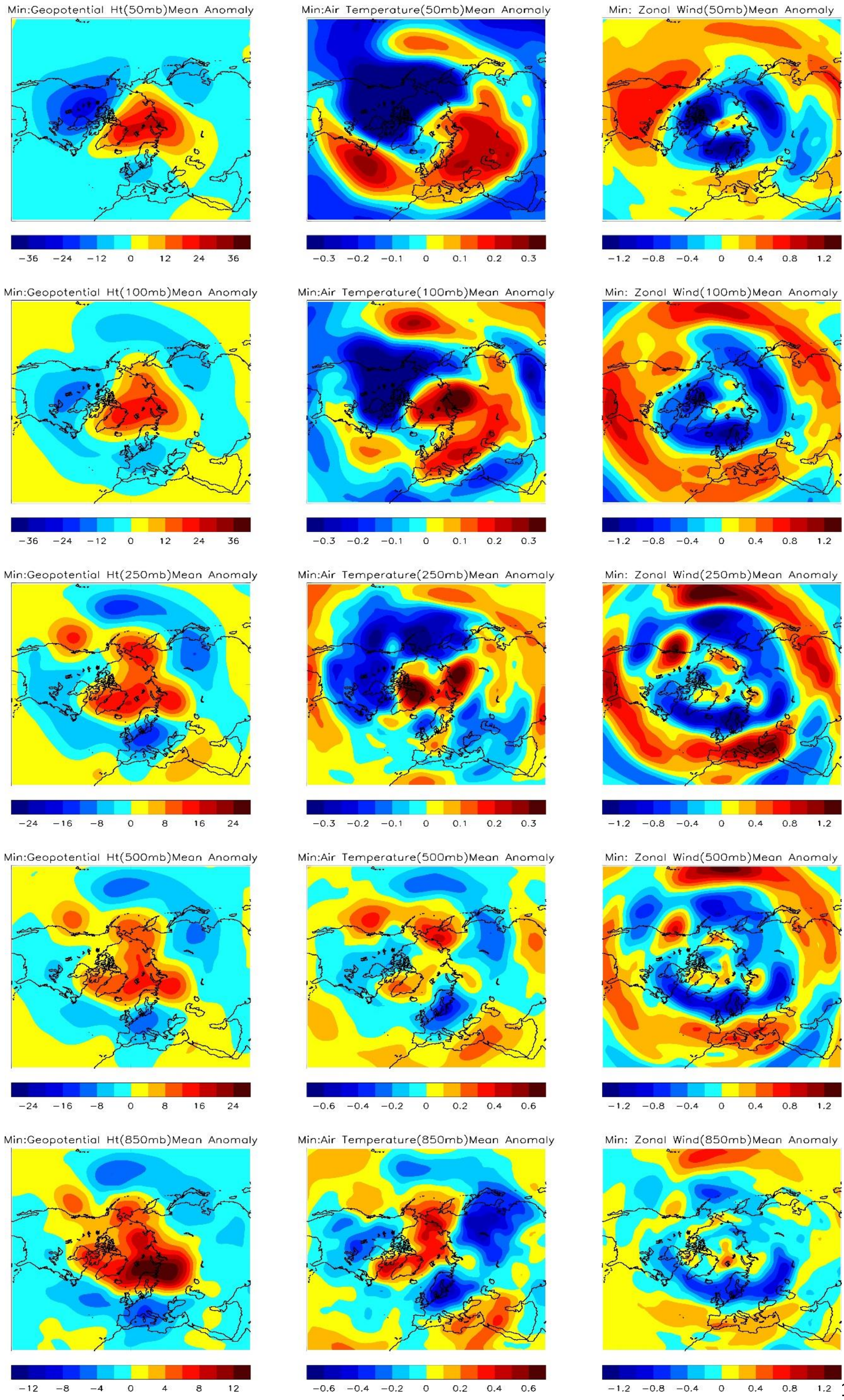
Min:Geopotential Ht(50mb)Mean Anomaly
−36 −24 −12 0 12 24 36
Min:Air Temperature(50mb)Mean Anomaly
−0.3 −0.2 −0.1 0 0.1 0.2 0.3
Min: Zonal Wind(50mb)Mean Anomaly
−1.2 −0.8 −0.4 0 0.4 0.8 1.2
Min:Geopotential Ht(100mb)Mean Anomaly
−36 −24 −12 0 12 24 36
Min:Air Temperature(100mb)Mean Anomaly
−0.3 −0.2 −0.1 0 0.1 0.2 0.3
Min: Zonal Wind(100mb)Mean Anomaly
−1.2 −0.8 −0.4 0 0.4 0.8 1.2
Min:Geopotential Ht(250mb)Mean Anomaly
−24 −16 −8 0 8 16 24
Min:Air Temperature(250mb)Mean Anomaly
−0.3 −0.2 −0.1 0 0.1 0.2 0.3
Min: Zonal Wind(250mb)Mean Anomaly
−1.2 −0.8 −0.4 0 0.4 0.8 1.2
Min:Geopotential Ht(500mb)Mean Anomaly
−24 −16 −8 0 8 16 24
Min:Air Temperature(500mb)Mean Anomaly
−0.6 −0.4 −0.2 0 0.2 0.4 0.6
Min: Zonal Wind(500mb)Mean Anomaly
−1.2 −0.8 −0.4 0 0.4 0.8 1.2
Min:Geopotential Ht(850mb)Mean Anomaly
−12 −8 −4 0 4 8 12
Min:Air Temperature(850mb)Mean Anomaly
−0.6 −0.4 −0.2 0 0.2 0.4 0.6
Min: Zonal Wind(850mb)Mean Anomaly
−1.2 −0.8 −0.4 0 0.4 0.8 1.2

Fig.S3. Same as Fig S2 for **Solar Min**, but the data is de-trended before the analyses. Plots are prepared using Met Office IDL (MIDL) software.

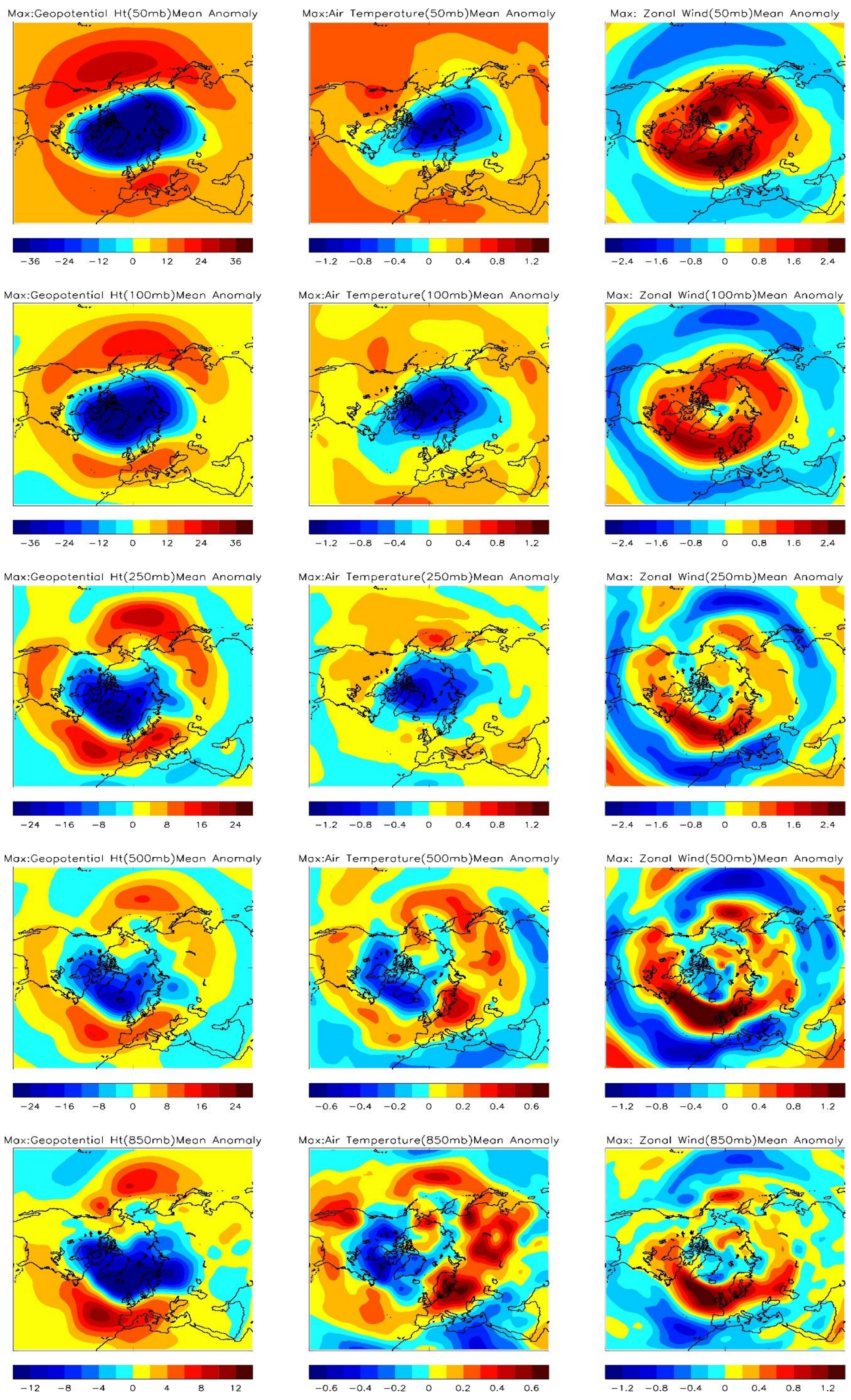
Max:Geopotential Ht(50mb)Mean Anomaly
−36 −24 −12 0 12 24 36
Max:Air Temperature(50mb)Mean Anomaly
−1.2 −0.8 −0.4 0 0.4 0.8 1.2
Max: Zonal Wind(50mb)Mean Anomaly
−2.4 −1.6 −0.8 0 0.8 1.6 2.4
Max:Geopotential Ht(100mb)Mean Anomaly
−36 −24 −12 0 12 24 36
Max:Air Temperature(100mb)Mean Anomaly
−1.2 −0.8 −0.4 0 0.4 0.8 1.2
Max: Zonal Wind(100mb)Mean Anomaly
−2.4 −1.6 −0.8 0 0.8 1.6 2.4
Max:Geopotential Ht(250mb)Mean Anomaly
−24 −16 −8 0 8 16 24
Max:Air Temperature(250mb)Mean Anomaly
−1.2 −0.8 −0.4 0 0.4 0.8 1.2
Max: Zonal Wind(250mb)Mean Anomaly
−2.4 −1.6 −0.8 0 0.8 1.6 2.4
Max:Geopotential Ht(500mb)Mean Anomaly
−24 −16 −8 0 8 16 24
Max:Air Temperature(500mb)Mean Anomaly
−0.6 −0.4 −0.2 0 0.2 0.4 0.6
Max: Zonal Wind(500mb)Mean Anomaly
−1.2 −0.8 −0.4 0 0.4 0.8 1.2
Max:Geopotential Ht(850mb)Mean Anomaly
−12 −8 −4 0 4 8 12
Max:Air Temperature(850mb)Mean Anomaly
−0.6 −0.4 −0.2 0 0.2 0.4 0.6
Max: Zonal Wind(850mb)Mean Anomaly
−1.2 −0.8 −0.4 0 0.4 0.8 1.2

Fig. S4. Same as Fig S2, but for **solar Max** and the data is de-trended before the analyses. Plots are prepared using Met Office IDL (MIDL) software.

a)

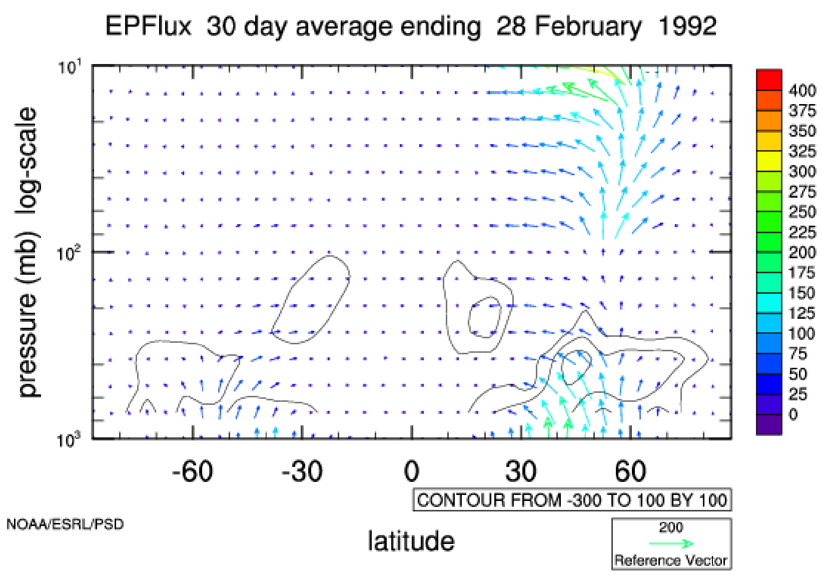

b)

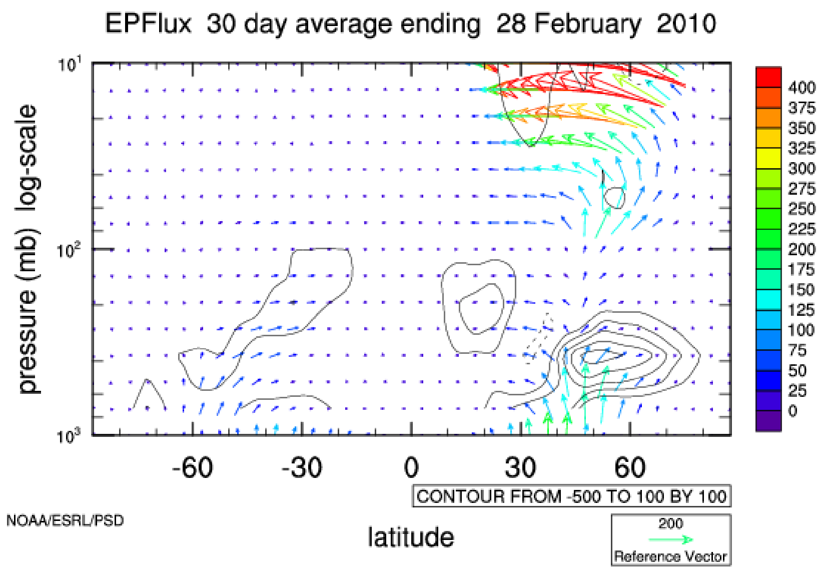

Fig. S5. EP-Flux computed from temperature and the wind (daily-averaged values) obtained from the NCAR/NCEP Reanalysis. Results of one Solar Max 1992 (a) and one Min (2010) (b) for one winter month (February) is presented. Contours show the value of EP Flux convergence/divergence. Solid lines show areas of EP-Flux convergence, and hence of westerly deceleration. For display purposes, the EP-Flux vectors above 100mb are multiplied by a scale factor 5. Plots generated using the link from NOAA/OAR/ESRL PSD, Boulder, Colorado, USA, from their website at (https://www.esrl.noaa.gov/psd/).

a)

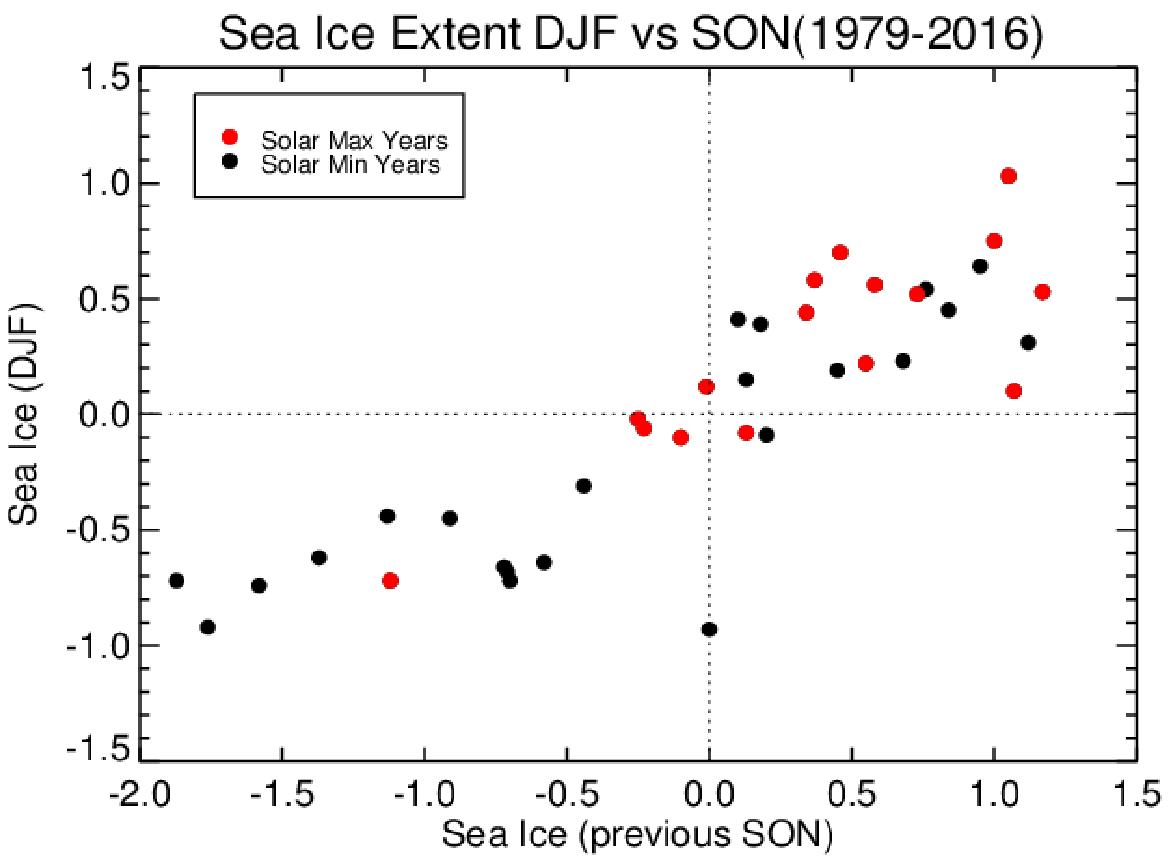
Sea Ice Extent DJF vs SON(1979-2016)
Solar Max Years
Solar Min Years
1.5
1.0
0.5
0.0
-0.5
-1.0
-1.5
Sea Ice (DJF)
-2.0
-1.5
-1.0
-0.5
0.0
0.5
1.0
1.5
Sea Ice (previous SON)

b)

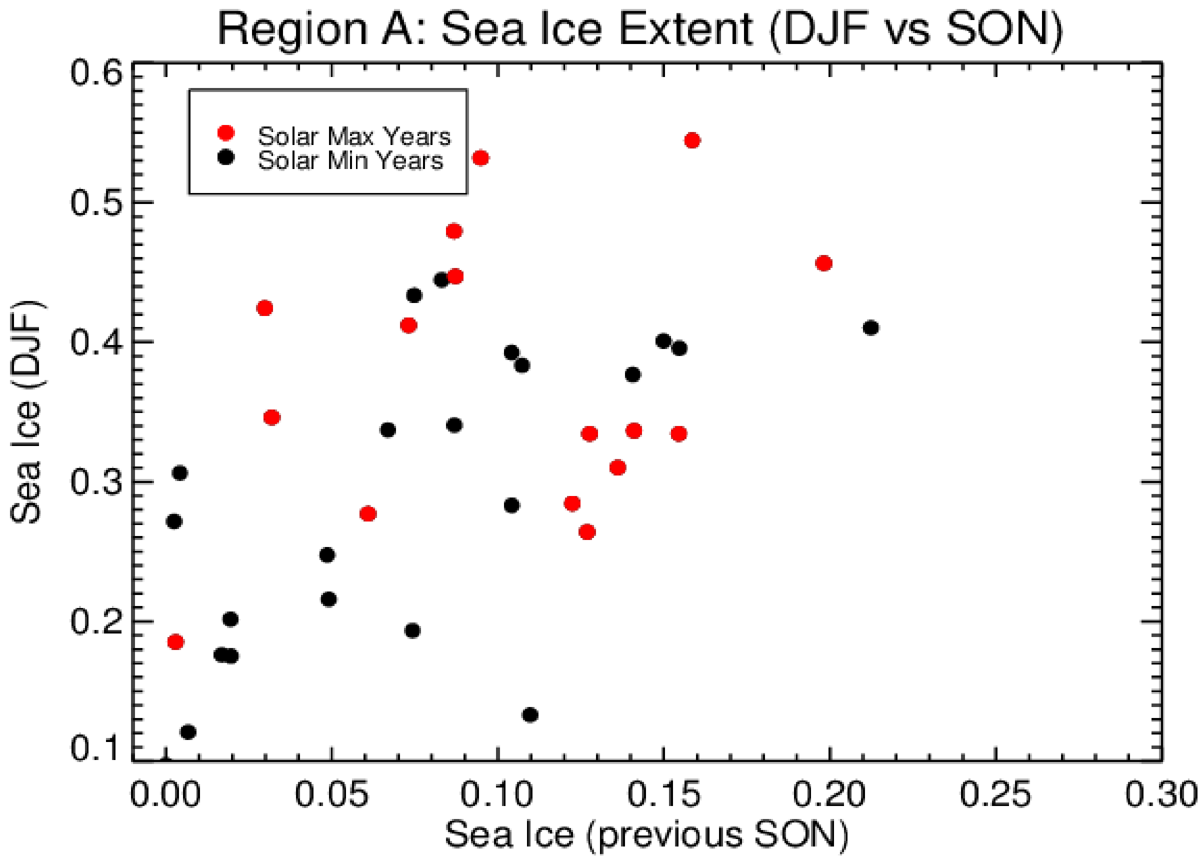
Region A: Sea Ice Extent (DJF vs SON)
Solar Max Years
Solar Min Years
0.6
0.5
0.4
0.3
0.2
0.1
Sea Ice (DJF)
0.00
0.05
0.10
0.15
0.20
0.25
0.30
Sea Ice (previous SON)

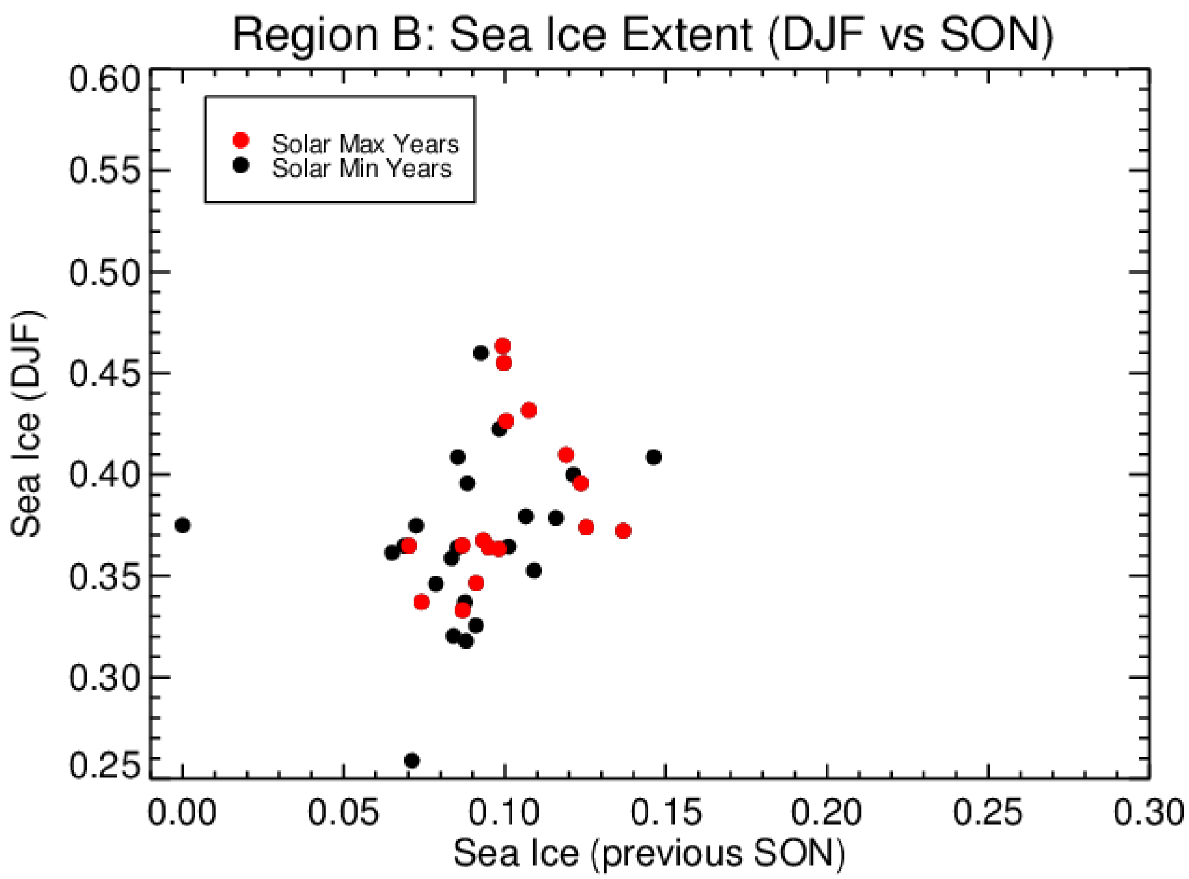
Region B: Sea Ice Extent (DJF vs SON)
Solar Max Years
Solar Min Years
Sea Ice (DJF)
Sea Ice (previous SON)
0.60
0.55
0.50
0.45
0.40
0.35
0.30
0.25
0.00
0.05
0.10
0.15
0.20
0.25
0.30

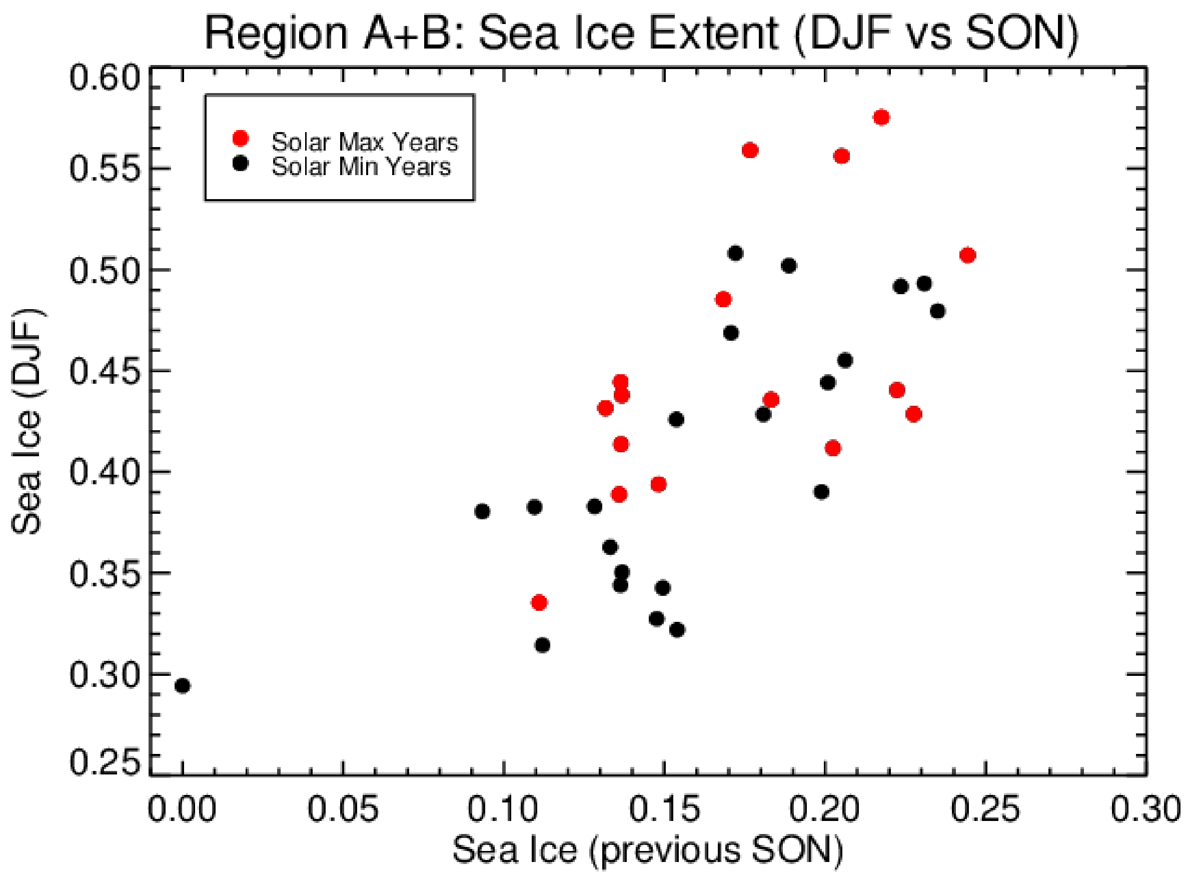
Region A+B: Sea Ice Extent (DJF vs SON)
Solar Max Years
Solar Min Years
Sea Ice (DJF)
Sea Ice (previous SON)
0.60
0.55
0.50
0.45
0.40
0.35
0.30
0.25
0.00
0.05
0.10
0.15
0.20
0.25
0.30

Fig.S6. Arctic Sea ice extent (million Sq-km) of solar Max and Min years, DJF against Sea ice extent of the previous season (SON). a) Anomaly plot for total sea ice extent; b) Sea ice extent for regions A, B and A+B respectively. Plots are generated using IDL software version 8.

a)

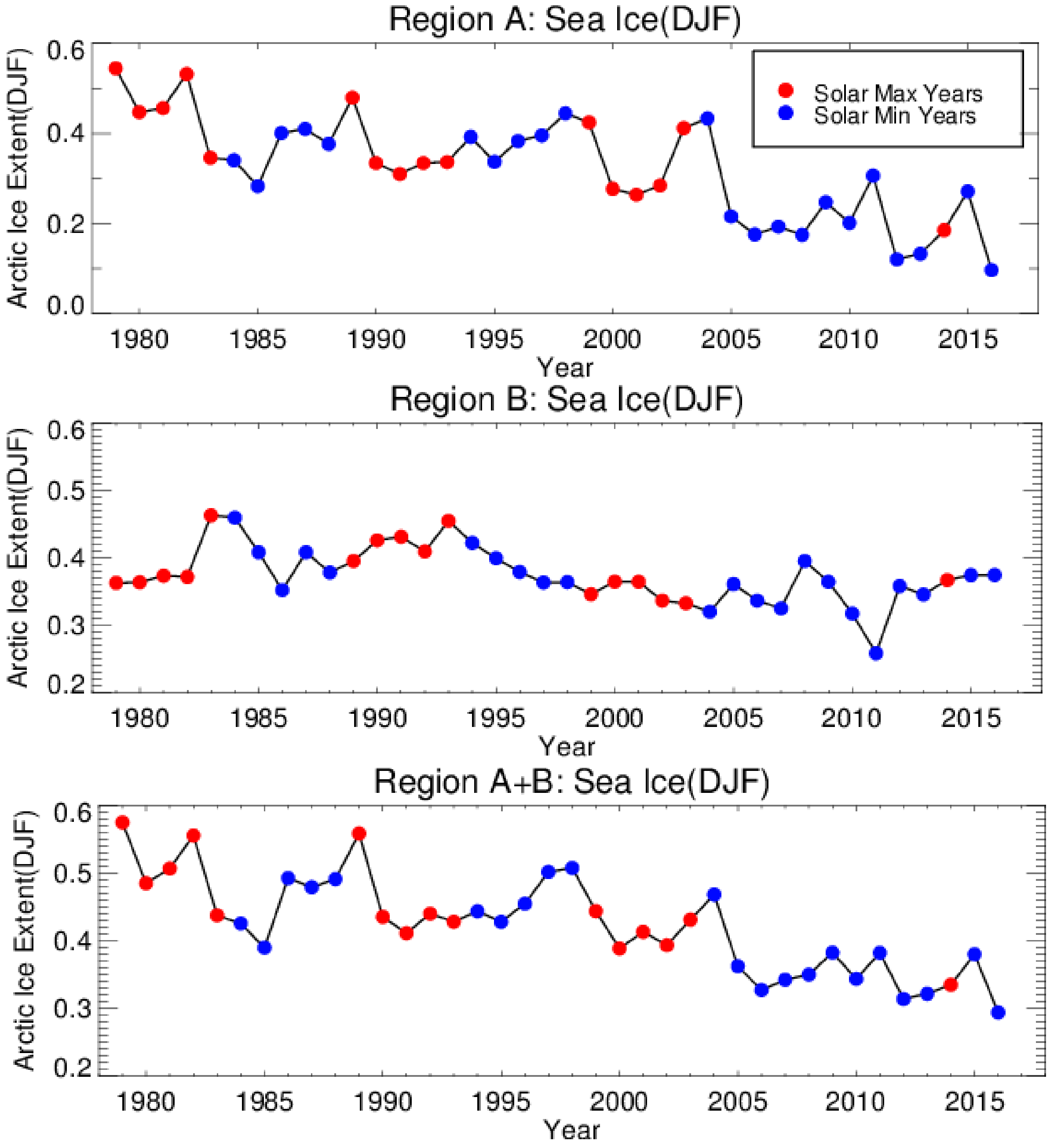

Region A: Sea Ice(DJF)
Solar Max Years
Solar Min Years
Arctic Ice Extent(DJF)
0.0
0.2
0.4
0.6
1980
1985
1990
1995
2000
2005
2010
2015
Year
Region B: Sea Ice(DJF)
Arctic Ice Extent(DJF)
0.2
0.3
0.4
0.5
0.6
1980
1985
1990
1995
2000
2005
2010
2015
Year
Region A+B: Sea Ice(DJF)
Arctic Ice Extent(DJF)
0.2
0.3
0.4
0.5
0.6
1980
1985
1990
1995
2000
2005
2010
2015
Year

b)

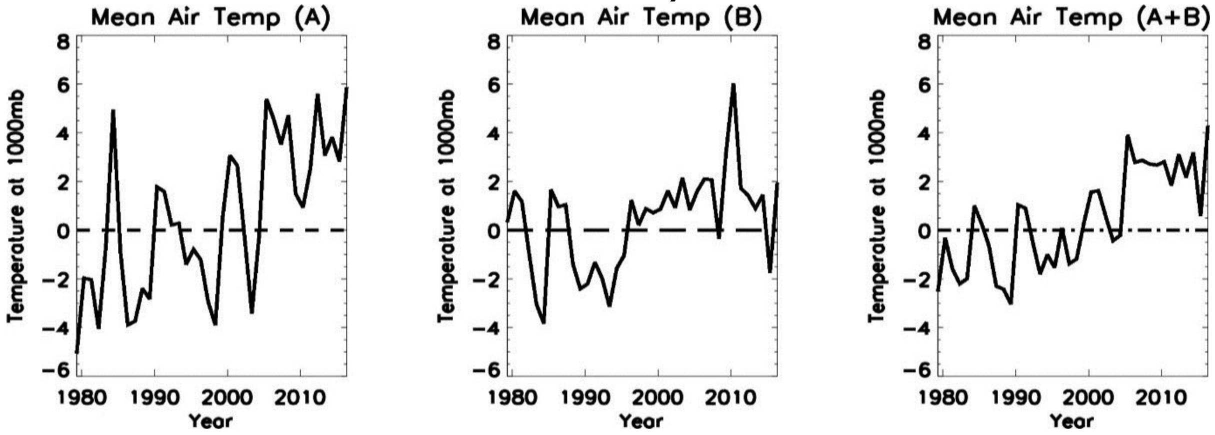

Mean Air Temp (A)
Mean Air Temp (B)
Mean Air Temp (A+B)
Temperature at 1000mb
-6
-4
-2
0
2
4
6
8
1980
1990
2000
2010
Year

Fig.S7. Time series plot for winter (DJF) Arctic sea ice extent (million Sq-km) (a), and mean air temperature (ºC) at 1000mb (b), in regions A, B and A+B. Plots are generated using IDL software version 8 (a) and Met Office IDL (MIDL) software (b).

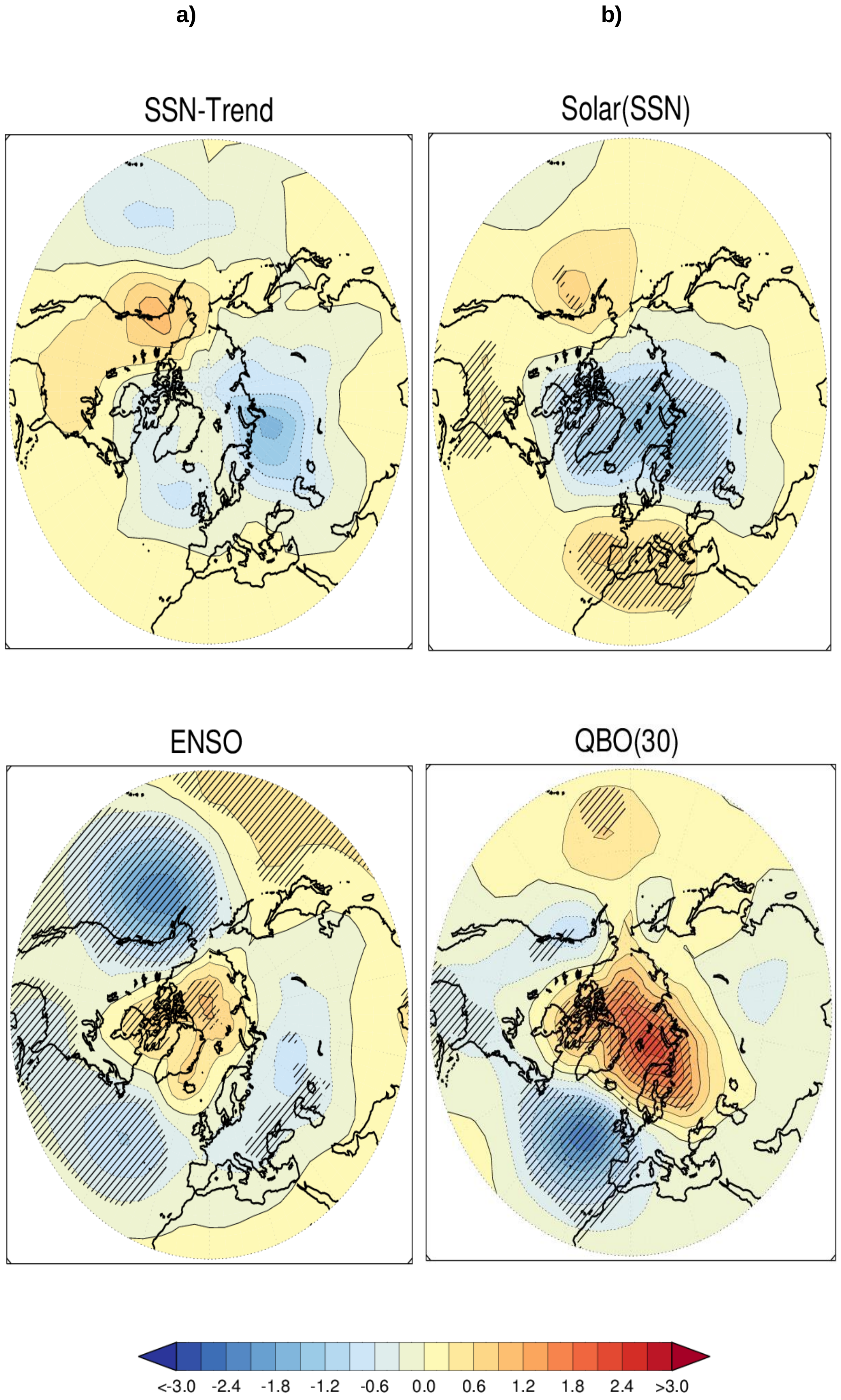

a)
b)
SSN-Trend
Solar(SSN)
ENSO
QBO(30)
<-3.0 -2.4 -1.8 -1.2 -0.6 0.0 0.6 1.2 1.8 2.4 >3.0

Fig.S8. The signal (hPa) in DJF, Hadley centre SLP data, obtained from a multiple linear regression (MLR) analysis over the 1979–2012 period. The results of 'SSN-Trend' that used the output from Fig. 6a (left) is presented in a). For b) independent indices used are SSN, ENSO, AOD (volcano) and QBO (30hPa). It is similar as Fig. 6, but the trend is not considered here. Signals are presented for SSN, ENSO and QBO as shown by subtitles. Negative contours are shown by dotted lines. Shaded regions are estimated significant at the 95% level using a two-sided Student's t-test. Note here the results of (Max-Min) are presented. Plots are prepared using IDL software, version 8.
